# Organoids with cancer stem cell-like properties secrete exosomes and HSP90 in a 3D nanoenvironment

**DOI:** 10.1371/journal.pone.0191109

**Published:** 2018-02-07

**Authors:** Takanori Eguchi, Chiharu Sogawa, Yuka Okusha, Kenta Uchibe, Ryosuke Iinuma, Kisho Ono, Keisuke Nakano, Jun Murakami, Manabu Itoh, Kazuya Arai, Toshifumi Fujiwara, Yuri Namba, Yoshiki Murata, Kazumi Ohyama, Manami Shimomura, Hirohiko Okamura, Masaharu Takigawa, Tetsuya Nakatsura, Ken-ichi Kozaki, Kuniaki Okamoto, Stuart K. Calderwood

**Affiliations:** 1 Department of Dental Pharmacology, Graduate School of Medicine, Dentistry, and Pharmaceutical Sciences, Okayama University, Okayama, Japan; 2 Advanced Research Center for Oral and Craniofacial Sciences, Okayama University Dental School, Okayama, Japan; 3 Department of Oral Morphology, Graduate School of Medicine, Dentistry, and Pharmaceutical Sciences, Okayama University, Okayama, Japan; 4 JSR Life Sciences Corporation, Tsukuba, Japan; 5 Department of Oral Pathology and Medicine, Graduate School of Medicine, Dentistry, and Pharmaceutical Sciences, Okayama University, Okayama, Japan; 6 Department of Oral Diagnosis and Dent-maxillofacial Radiology, Graduate School of Medicine, Dentistry, and Pharmaceutical Sciences, Okayama University, Okayama, Japan; 7 Radio Isotope Research Center, Okayama University Dental School, Okayama, Japan; 8 Division of Cancer Immunotherapy, Exploratory Oncology Research and Clinical Trial Center, National Cancer Center, Kashiwa, Japan; 9 Department of Radiation Oncology, Beth Israel Deaconess Medical Center, Harvard Medical School, Boston, MA, United States of America; Università degli Studi della Campania "Luigi Vanvitelli", ITALY

## Abstract

Ability to form cellular aggregations such as tumorspheres and spheroids have been used as a morphological marker of malignant cancer cells and in particular cancer stem cells (CSC). However, the common definition of the types of cellular aggregation formed by cancer cells has not been available. We examined morphologies of 67 cell lines cultured on three dimensional morphology enhancing NanoCulture Plates (NCP) and classified the types of cellular aggregates that form. Among the 67 cell lines, 49 cell lines formed spheres or spheroids, 8 cell lines formed grape-like aggregation (GLA), 8 cell lines formed other types of aggregation, and 3 cell lines formed monolayer sheets. Seven GLA-forming cell lines were derived from adenocarcinoma among the 8 lines. A neuroendocrine adenocarcinoma cell line PC-3 formed asymmetric GLA with ductal structures on the NCPs and rapidly growing asymmetric tumors that metastasized to lymph nodes in immunocompromised mice. In contrast, another adenocarcinoma cell line DU-145 formed spheroids in vitro and spheroid-like tumors in vivo that did not metastasize to lymph nodes until day 50 after transplantation. Culture in the 3D nanoenvironment and in a defined stem cell medium enabled the neuroendocrine adenocarcinoma cells to form slowly growing large organoids that expressed multiple stem cell markers, neuroendocrine markers, intercellular adhesion molecules, and oncogenes in vitro. In contrast, the more commonly used 2D serum-contained environment reduced intercellular adhesion and induced mesenchymal transition and promoted rapid growth of the cells. In addition, the 3D stemness nanoenvironment promoted secretion of HSP90 and EpCAM-exosomes, a marker of CSC phenotype, from the neuroendocrine organoids. These findings indicate that the NCP-based 3D environment enables cells to form stem cell tumoroids with multipotency and model more accurately the in vivo tumor status at the levels of morphology and gene expression.

## Introduction

Two-dimensional (2D) monolayer culture carried out using specially prepared polystyrene dishes has revolutionized modern biology, allowing precise and reproducible experimentation. The modified plastic is able to bind fibronectin and vitronectin from serum, thus offering a surface for cell adhesion [1]. However, the 2D culture conditions can enhance integrin signaling and thus mask many aspects of cellular physiology [2]. In contrast, three-dimensional (3D) cell culture can replicate some aspects of the physiological or pathological milieu, such as the intratumoral microenvironment [3] and thus be more suitable for some aspects of cancer research. For instance, the mesenchymal transition of non-small cell lung carcinoma (NSCLC) cell lines was much more efficiently induced on 3D cell culture condition than under 2D conditions [4]. Alteration of epithelial-mesenchymal transition (EMT) properties of lung adenocarcinoma cell line A549 was efficiently quantified using the NanoCulture Plate (NCP)-based 3D cell culture system [5]. The NCPs are cell/tissue culture plates with patterned nanoscale grids that restrict cells from sprawling on the base and enables cells to migrate more readily than in monolayer cell culture [6]. Cells migrate from one scaffold to another scaffold on the grid on the NCPs more actively than cells cultured on the 2D plates, a property reminiscent of tumor cell behavior. The increased migration and reduced attachment of cells to the NCPs also enable them to form 3D cell aggregates. Other advantages of the NCP are that it does not require gel materials [6] and cells cultured on NCPs have higher rates of proliferation comparable with those cultured on 2D plates. The NCP-based cell-culture system can also be useful for selection of malignant cancer cells that can grow under anchorage-independent conditions as also shown in soft-agar. The NCP-based 3D cell-culture method is thus suitable for assessing key phenotypic changes in tumor progression. Indeed, an EMT quantification system utilizing the NCPs contributed to a discovery of a novel EMT inhibitor [5]. However, in addition to EMT, the treatment of cancer cells is a further unsolved risk for patient death.

Regression versus progression of tumors depends on a balance between immunity [7, 8], the tumor microenvironment [9], and the strength of carcinogenic stimuli [10–12]. Tumors are often resistant to immunity [13], chemotherapy [14, 15] and radiation therapy [16] due to clonal evolution which generates multiple variant genotypes and thus novel phenotypes in cancer cells [17–20]. Gene expression of the stress resistance protein HSP family is elevated and potentially responsible for resistance in such advanced cancer cells [21–27]. The tumor microenvironment consists of cancer cells including cancer stem cells (CSC) [28–30], cancer-associated fibroblasts (CAF) [31, 32], mesenchymal stem cells (MSC) [33–35], immune / inflammatory cells [36–38], and vascular endothelial cells [39]. These types of cells within the tumor microenvironment communicate via cell surface molecules, secretory factors and/or extracellular vesicles and play key roles in tumor progression [40, 41]. Hypoxic microenvironments have been also shown to arise in tumors *in vivo*, due the cells outgrowing the local microcirculation [42–44]. Hypoxia then leads to induction of transcription factors, HIF1α and HIF2α, shown to target multiple genes, including stem cell-related genes such as OCT4/POU5F1, IL-6, hTERT, Notch, Notch ligand DLL4, TGFα and ABC transporter genes [42–44]. Therefore, we hypothesized that hypoxia within the tumor microenvironment could enhance stem cell phenotypes. The interior of cell aggregates (tumoroids) formed by strong intercellular adhesion can also become highly hypoxic and thus stemness may potentially be induced in vitro in non-adherent cell culture and in vivo in tumors. By contrast, the frequently used 2D in vitro cell culture system is directly exposed to oxygen and does not model the conditions of in vivo hypoxic tumors. In the present study, we have examined altered expression of SC markers and identify genes in the highly hypoxic enlarged tumoroids (cell aggregates) generated in the NCP-based 3D culture system.

Stem cells were first found in hematopoietic cells and thus designated hematopoietic stem cells (HSCs) [45]. Unequivocal proof of HSCs has given way to the prospective isolation of tissue-specific stem and progenitor cells [34, 35, 46]. Tumors may often originate from the transformation of normal stem cells, and cancer cells may include sub-populations of CSCs [28, 30, 45]. Paradoxically, teratoma formation in experimental animals is one of the features of induced pluripotent stem (iPS) cells [47, 48]. Indeed, an increased in expression of normal SC markers has been found in cancer stem cells [34, 49, 50]. Currently defined characteristics of CSCs are cell aggregates/spheroid formation, tumor initiation, a relatively slow cell cycle or entrance into dormancy, exhibiting chemo-resistance, SC marker expression, and pluripotency [28–30, 51–53]. Dormant cancer cells within subclones can survive chemotherapy while proliferating subclones are relatively more chemotherapy sensitive. Thus, tumors can relapse due to cells surviving after treatment and re-established subclonal diversity [52]. Therefore, in the present study, we examined whether 3D cell culture conditions could induce characteristics of CSCs including dormancy of the evolved castration-resistant adenocarcinoma cells.

Prostate CSCs were firstly identified by Collins et al in 2005 [54]. Several biomarkers of CSCs in prostate and other types of cancers have been shown, including CD133/PROM1 [54–56], CD326/EpCAM [56], a chemokine receptor CXCR4 [55, 57], aldehyde dehydrogenase (ALDH) activity [58], Integrin α2 and β1 [54], stimulus-independent PTEN/Akt/PI3K signaling [59], protein fucosylation [60] and CD44 alternative splicing variants (CD44v) [61, 62]. Expression of many of these molecules is associated with a poor prognosis in patients [63–65]. Although CD44 has been used as a biomarker of CSCs, it has been shown more recently that CD44 variants could be expressed in such cells. The CD44 transcripts expressed in most cells are known as CD44 standard (CD44s) and do not contain sequences from an interior variable region and does promote EMT [66]. Epithelial splicing regulatory proteins (ESRP1 and ESRP2) have been shown to regulate epithelial cell-type-specific splicing [67], including CD44v / CD44s switching regulated by ESRP1 in breast cancer cells [66]. The expression of ESRP2 was shown to be suppressed by TGF-β through induction of ZEB1 and ZEB2 [68]. In addition, growth factor-dependent and independent activations of receptor tyrosine kinases (RTKs) have been shown to stimulate the PI3K-Akt pathway-dependent EMT [69]. We recently showed that NCPs induce cellular aggregation (tumoroids) mediated by the intercellular adhesion molecule E-cadherin *in vitro*, whereas TGF-β signaling oppositely repressed cellular aggregation via promotion of EMT on the NCP-based 3D cell culture system [5]. Thus, EMT promotes transformation and migration of cancer cells, whereas inhibition of EMT increases intercellular adhesion and thus promotes cellular aggregation. Under these conditions, drug-resistant CSCs can be concentrated and initiate recurrence- a phenomenon called the “EMT-CSC paradox”. The EMT-CSC paradox is contradicted to the model that EMT induces CSC phenotypes [14, 70]. The complicated relationship between the EMT and CSC phenotypes has not been unequivocally resolved yet. In the present study, we have aimed to clarify the EMT-CSC paradox.

To define the nature of cell aggregates formed on NCPs, we first examined sixty seven cell lines cultured under these conditions and classified their varied morphologies. Such morphologies included: spheres (a regular three-dimensional object in which every cross-section is a circle), spheroids (similar form to spheres, but not quite the same. Distorted sphere-like structure), grape-like aggregation (GLA), other types of aggregation, and monolayer sheets. We also showed that expression of stem cell marker genes and oncogenes were increased in the cell aggregates formed by castration-resistant neuroendocrine adenocarcinoma cells growing under the 3D conditions.

## Materials and methods

### Cells

PC-3, MCF7, T47-D, DLD-1, SW480, MiaPaCa-2, BT474, MDA-MB-231, COLO205, HT29, SW620, BT-20, BxPC-3, Capan-1, Capan-2, COLO201, HCC1806, HCC1954, HCT-116, Hep3B, Hs578T, NCI-H1650, NCI-H1975, NCI-H1993, NCI-H2030, NCI-H23, NCI-H441, OVCAR-3, PLC/PRF/5, RKO, SNU-16, SNU-5, WiDr, Calu-1, HeLa, SK-BR-3, ZR-75-30, BT549, HeLa, ACHN, MDA-MB-435S, DU-145, LNCaP, RWPE1, CAL 27, FaDu, and SK-OV-3 were obtained from American Tissue Culture Collection. MKN-45, MKN-74, huH-1, U251, HSC-3, HSC-3-M3, Colon26, and 3T3L1 were obtained from JCRB Cell Bank at National Institutes of Biomedical Innovation, Health, and Nutrition. HLE, HLF, JHH-2, JHH-5, JHH-6, JHH-7, ASPC-1, and SUIT-2 were obtained from National Cancer Center. A549, Panc-1, HepG2, RBE, and Li-7 were obtained from Riken Cell Bank. RT7 was provided by Dr. Nobuyuki Kamata at Hiroshima University. Each cell line was maintained and cultured in the recommended medium unless otherwise specified. LuM1 and NM11 were maintained as described previously [71]. Characteristics of prostate adenocarcinoma cell lines were summarized in S1 Table.

### NCP-based 3D cell culture and stemness induction

For 3D culture, cells were cultured in NanoCulture Plates (NCPs) (MBL Corporation, Nagoya, Japan) as described [5]. For stem-cell conditions, cells were cultured in mTeSR1 medium (Stem cell technologies) containing LiCl (1 mM), basic FGF (100 ng/ml), TGF-β (23.5 pM), GABA (1 mM), insulin (4 μM), transferrin (0.137 μM), β-mercaptoethanol (0.1 mM), cholesterol (1.12 μM), lipids, and BSA in the DMEM/F12 basal medium [72, 73]. Photomicrographs of cell aggregates were taken by using a microscope IX71 with a DP72 camera (Olympus).

### Quantification of hypoxic cell aggregations

As described [5]. Cells were seeded at a concentration of 1,000 to 10,000 cells/well in 96-well NCPs (MBL Corporation, Nagoya, Japan). For time-lapse analysis, cells were monitored using Array Scan High Content Screening (HCS) System (Thermo, Waltham, MA). For hypoxia analysis, a hypoxia probe Lox-1 (MBL Corporation, Nagoya, Japan) was added at a final concentration of 2 μM the day before the measurement. The fluorescence intensity of each pixel (μm^2^) and per aggregations of cells was determined using a filter set for Texas Red. Fluorescent areas greater than 100 μm^2^ were counted as cellular aggregations, while those less than 100 μm^2^ were not. A single well-area of a 96-well plate was sectionized to 9 fields, and the area of 1 field is 2,632 μm^2^. For counting cellular aggregations, fluorescent intensity and area (μm^2^ = pixel) of each aggregation in the entire cell population or per field were calculated. For time-lapse analysis, 1,000 PC-3 cells were seeded per well in a 96-well NCP in RPMI containing 5% FBS or mTeSR1 for 7 days.

### Flow cytometry

Antibodies were labeled using Phycoerythrin labeling kit NH2 (Dohjindou, Japan). Cells were cultured in 75 cm^2^ tissue culture flask and detached by using trypsin/EDTA. Detached cells were centrifuged at 1,000 rpm for 5 min. Cells were suspended in PBS(-) at a concentration of 10^6^ cells/ml. To a 100 μl of cells in PBS, 3 μl of antibodies were added and incubated for 1 hour at RT. Then, 1 ml of PBS(-) was added to cell-antibody complex and centrifuged at 1,000 rpm for 5 min. The supernatant was aspirated with a pipette and Bemcot (Asahi Kasei, Japan). This wash step was repeated. Flow cytometry was carried out using BD Accuri C6 (BD Bioscience). An anti-human CD44 v9 mAb (clone RV3) Phycoerythrin (PE)-labeled were obtained from Cosmo Bio. An anti-PSA antibody IgG1κ was prepared by immunizing mouse with human PSA (Scripps laboratories. P0725) and labeled with PE.

### Splicing variant analysis (conventional RT-PCR)

As described [74]. The 1 μg of total RNA was denatured at 65°C for 5 min, and then mixed with 5x RT buffer, 25 pmol random 9mer, 5 pmol oligo dT primer adapted with M13 primer M4 seq, dNTPs (final 1 mM), 40 unit RNase inhibitor and 100 unit ReverTra Ace RTase (Toyobo, Osaka) in 20 μl reaction. The cDNA was synthesized by incubating the mixture at 30°C for 10 min, at 42°C for 30 min, and at 99°C for 5 min. For PCR, 2.5 μl of the cDNA was mixed with 10x PCR buffer, 2.5 μl of 2 mM dNTPs, forward and reverse primers (final 0.2 μM each), and 1.25 unit Blend Taq-Plus (Toyobo, Osaka) in total 25 μl. The mixture was denatured at 94°C and PCR was carried out by 40 cycles of 94°C for 30 secs, 55°C for 30 secs, and 72°C for 1 min, followed by a final extension at 72°C for 5 min. The 10 μl of PCR products were mixed with 6x loading dye and used for electrophoresis in 11 cm x 10 cm 2% agarose gel at 100 V for 1.5 hrs., and stained in 0.5 μg/ml EtBr for 30 min, washed in ddH_2_O for 30 min. Primers for *CD44s/v* [74] and *ACTB* were listed in S2 Table.

### Real-time qRT-PCR

As described [35]. cDNA was synthesized by using ReverTra Ace (Toyobo, Osaka, Japan). Real-time PCR was carried out by using SYBR Green PCR master mix plus (Toyobo) or iQ cyber (BioRad). Primers for *GAPDH*, *ESRP1*, *ESRP2* [68], *ECAD/CDH1*, *CD133/PROM1*, *CD44s* and *CD44v9* [75] were listed in S2 Table.

### Gene expression profiling

As described [23, 35]. RT^2^ Profiler PCR array Human Cancer Stem Cells (SABiosciences) was used, and the data were analyzed by using RT^2^ profiler PCR array data analysis ver 3.5 (SABiosciences). For building block analysis, the genes were categorized into 6 categories: stem cell and pluripotency; hippo signaling; intercellular adhesion, anti-EMT, and Notch-delta signaling; tyrosine kinases; pro-EMT; immune and hemangiogenic. The genes expressed at the highest level in a condition as compared to the other conditions were counted to construct building blocks.

### Whole cell lysate

As described [5, 76]. Cells were cultured until being sub-confluent, confluent or over-confluent on 2D culture plates for 2 to 5 days, or on 35-mm NCPs for 8 to 12 days. Media were changed every 3 days. Cells were washed with PBS (-), treated with 150 to 200 μl/dish of a 1x RIPA buffer containing 1% NP-40, 0.1% SDS, and 0.5% deoxycholate, and protease inhibitors in PBS (-), and collected by using a cell scraper. Cells were further lysed by a 25G needle-syringe for 10 strokes and then incubated for 30 min on ice. The lysate was centrifuged at 12,000 x g for 20 min at 4°C and the supernatant was used as a whole cell lysate (WCL). The WCL was diluted 10-fold and protein concentration was measured by using micro BCA protein assay system (ThermoFisher Scientific).

### Preparation of exosomes

Cells cultured were washed with Hanks’ balanced salt solution (HBSS), and then further cultured in serum-free medium for 2 days. Cell culture supernatant was centrifuged at 2,000 x g for 30 min at 4°C to remove detached cells. The supernatant was then centrifuged at 10,000 x g for 30 min at 4°C to remove cell debris. The supernatant was concentrated by using an Ultra-15 Centrifugal Filter Devices for MW. 100,000 (Amicon). The concentrate was applied to a polymer method using Total Exosome Isolation (ThermoFisher Scientific). The exosome fractions were eluted in 100 μl PBS (-). The filtrated non-exosome fraction (< 100 kD) was applied to Ultra-15 Centrifugal Filter Devices for MW. 10,000 (Amicon) and centrifuged at 10,000 x g for 10 min to concentrate the fraction to less than 400 μl and then a protease inhibitor cocktail was added. For protein assay, 10x RIPA buffer containing 10% NP-40, 1% SDS, and 5% deoxycholate in PBS (-) and an EDTA-free protease inhibitor cocktail (Sigma) were added to the exosome fraction, incubated on ice for 15 min and applied to micro BCA protein assay system (ThermoFisher Scientific).

### Transmission electron microscopy (TEM)

A 400-mesh copper grid coated with formvar/carbon films was hydrophilically treated. The exosome suspension (5 to 10 μl) was placed on Parafilm, and the grid was floated on the exosome liquid and left for 15 min. The sample was negatively stained with 2% uranyl acetate solution for 2 min. exosomes on the grid were visualized with 20,000 times magnification with an H-7650 transmission electron microscope (Hitachi, Tokyo, Japan) at Central Research Laboratory, Okayama University Medical School.

### Western blotting analysis

As described [76]. Each protein sample per 3 x 10^5^ cells was used for analysis of exosome, per 1 x 10^5^ cells was used for analysis of non-exosome cell culture supernatant, and per 2 x 10^4^ cells was used for analysis of cell lysates. For analysis of CD9 and EpCAM, protein samples were mixed with an SDS sample buffer without any reducing agent and boiled. Otherwise, protein samples were mixed with the SDS sample buffer with β-mercaptoethanol, boiled, separated by SDS-PAGE in 4–20% TGX-GEL (BioRad) or 7.5% poly-acrylamide gel, and transferred to PVDF membranes by using a semi-dry method or tank method. Blocking and antibody reactions were done in a blocking buffer containing 5% skim milk (Wako) or ECL Blocking Agent (GE Healthcare) in Tris-buffered saline containing 0.05% Tween 20 (TBS-T). The membrane was blocked for 1 h with shaking at RT. Each membrane was washed three times with TBS-T for 10 min with shaking at RT and then incubated overnight with shaking at 4˚C with primary antibodies: either mouse anti-CD9 (1:1,000, MBL), mouse anti-EpCAM (1:1,000, VU1D9, CST), rabbit anti-Vimentin (1:1,000, D21H3, CST), rabbit anti-E-cadherin (1:1,000, 24E10, CST). Afterwards, the membranes were incubated for 1 h with shaking at RT with horseradish peroxidase (HRP)-conjugated secondary antibodies; either anti-mouse IgG (1:10,000, CST) or anti-rabbit IgG (1:10,000, GE Healthcare). Washes after antibody reactions were done on a shaker within TBS-T at RT three times for 5 min and three times for 10min. Alternatively, membranes were incubated with HRP-conjugated mouse anti-β-actin (1:5,000, Wako) antibodies for 1 h with shaking at RT. Blots were visualized with ECL Plus western blotting substrate (Pierce).

### Cell proliferation and viability

Cells were seeded at a concentration of 2.3x10^4^ cells/well in a 24-well culture plate (2D) or 24-well NCP (3D). Number of cells at day 2 to 14 were counted by using Countess automated cell counter (Invitrogen). For detachment of adherent cells from the plates and for separation of cell aggregates, cells were treated with 0.25% Trypsin-EDTA solution. For photomicrography, a Floid cell imaging station (Thermo Fisher Scientific) or phase contrast microscope (Olympus CKX 53) were used. We used the following formula. Doubling Time = duration x log(2) / log(Final Concentration) − log(Initial Concentration).

### Immunohistochemistry (IHC)

For 2D IHC, cells were cultured on coverslips coated with collagen and fixed in 4% paraformaldehyde in PBS (-) pH 7.4 for 10 min at RT. Fixed cells were permeabilized for 10 min in PBS containing 0.1% Triton X-100. Endogenous peroxidases were blocked for 30 min in 1.5% H_2_O_2_ for 30 min. For blocking non-specific reactions of primary antibodies, cells were incubated for 20 min at RT in serum-free protein blocking reagent (Dako, Tokyo, Japan). Hereinafter, primary antibodies were used at 4˚C for 16 hrs. The applied primary antibodies were follows: anti-Chromogranin A antibody (ab15160, 1:400, abcam, Cambridge, UK), anti-Vimentin antibody (ab8978, 1:1000, abcam, Cambridge, UK), anti-EpCAM antibody (ab71916, 1:200, abcam, Cambridge, UK), anti-Synaptophysin antibody (ab32127, 1:1000, abcam, Cambridge, UK) and anti-NCAM antibody (ab9018, 1:1000, abcam, Cambridge, UK). To react with the primary antibodies, peroxidase-labeled polymer (Simple Stain MAX-PO (MULTI) ®, Nichirei Biosciences, Tokyo, Japan) was allowed to react for 30 min at RT and was developed using DAB (Liquid DAB + Substrate Chromogen System, Dako, Tokyo, Japan) for visualizing of immunohistochemical reactions. Samples were then counterstained with hematoxylin. For negative control, the same protocol was performed under omission of the primary antibody. For analysis of immunostaining were counted in five areas chosen from the selected regions at x200 magnification. One hundred cells were counted in each area and positive cell ratio was calculated.

For 3D IHC, cells were cultured for 11 days and then cell aggregates were fixed in 4% paraformaldehyde in PBS (-) for 8 min. The cell aggregates were washed with PBS (-) for 5 min 3 times and embedded in paraffin at Central Research Laboratory, Okayama University Medical School. Antigen retrieval was performed with Histo/zyme (Diagnostic BioSystems, CA) for 5 min, and sections were incubated with blocking solution (1% BSA and 10% normal goat serum) for 1 h and then with primary antibodies; mouse anti-EpCAM (#2929, CST, 1:200), rabbit anti-Vimentin (#5741, CST, 1:100), and rabbit anti-E-cadherin (#3195, CST, 1:100). Alexa Fluor Plus secondary antibodies (Thermo Scientific, Waltham, MA) were used. Fluorescent images were taken by using Keyence microscopy system and modified by haze reduction programs.

### In vivo tumorigenicity and metastasis

This study was carried out in strict accordance with the recommendations in the Guide for the Care and Use of Laboratory Animals of the Japanese Pharmacological Society. The protocol was approved by the Committee on the Ethics of Animal Experiments of the Okayama University (Permit Number: OKU-2016219). All surgery was performed under sodium pentobarbital anesthesia, and all efforts were made to minimize suffering. Cells were pre-cultured on 2D plates or 3D NCP and detached with Trypsin/EDTA. Cells (1 x 10^6^) were subcutaneously transplanted on the backs of SCID mice. The major and minor axis of developed tumors were measured.

### Statistical analysis

All data are expressed as the mean ± standard deviation unless otherwise indicated. Statistical analysis was performed using ANOVA. Differences between groups were rated significant at values of P < 0.05 or P < 0.01.

## Results

### Classification of cell aggregates

**Fig 1:**
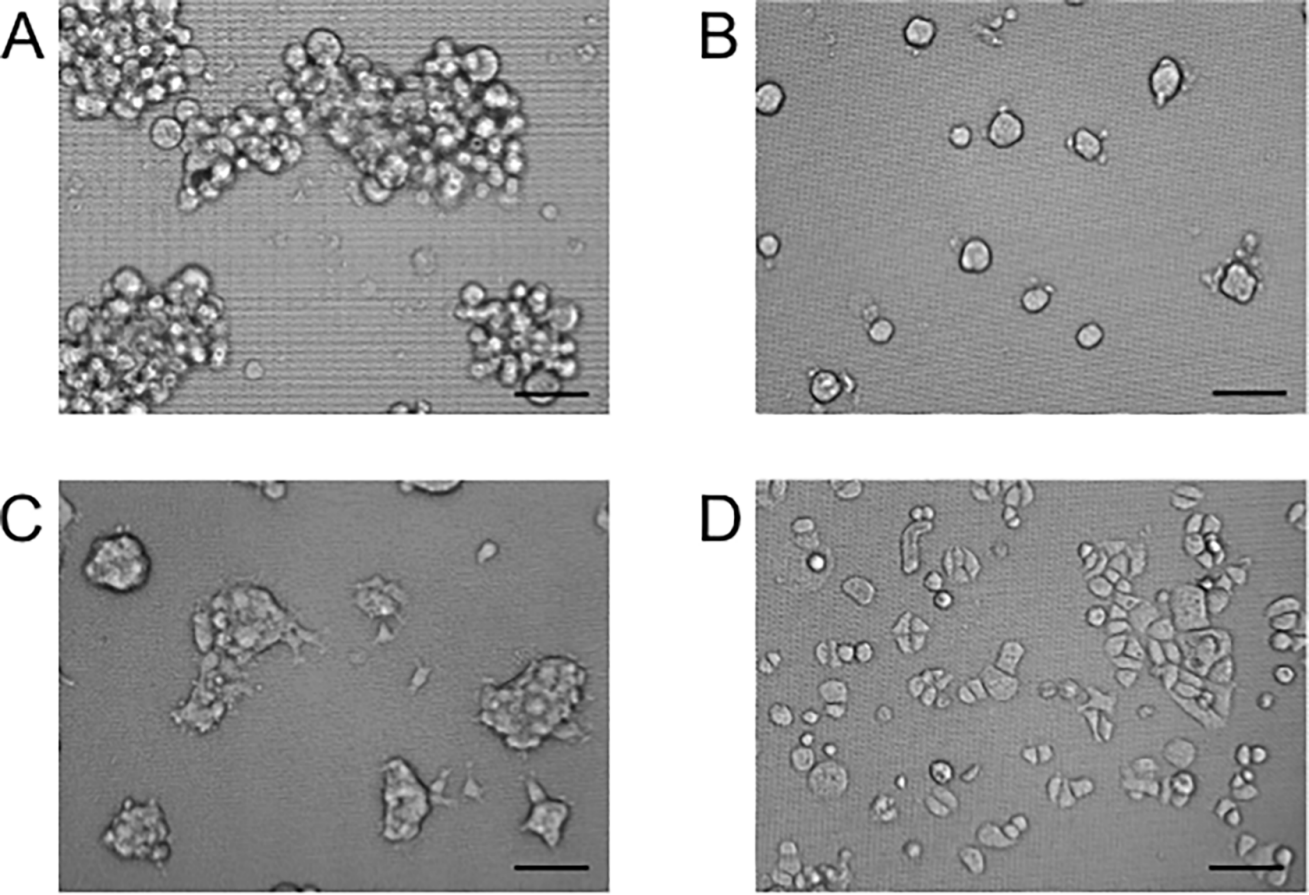
Representative morphologies of the various types of cell aggregates formed on NanoCulture Plates (NCP). (A) Grape-like aggregation (GLA) of PC-3 cells formed in F12K medium with 10% FBS. (B) Spheroids of CAL 27 cells formed in DMEM with 10% FBS. (C) Cell aggregation of FaDu cells formed in DMEM with 10% FBS. (D) Epithelial monolayer sheet of RT7 cells formed in KGM2 medium. Scale bar, 100 μm. The morphological classification of 67 cell lines on NCP is shown in Table 1.

Many types of cells, including tumor cells, are able to form multicellular aggregates on low-attachment plates or NanoCulture plates (NCPs) *in vitro* [4–6]. In the present study, we showed that not only spheroids/spheres but also new types of cellular aggregates could be formed on the NCPs. Among the 67 types of cell lines that we cultured on NCPs, 8 cell lines formed grape-like aggregation (GLA) (Fig 1A), 49 cell lines formed sphere or spheroids (Fig 1B), 8 cell lines formed other types of aggregation of cells (Fig 1C), and 3 cell line formed monolayer epithelial-like sheets (Fig 1D) (Table 1).

**Table 1:** Classification of the morphologies of cellular aggregations.

Morphology	Name of cell lines	Number of cell lines
**Spheroid**	A549, AsPC-1, BT20, BT549, BxPC-3, CAL 27, Calu-1, Capan-1, Colon-26, Colo-205, DU-145, HCC1806, HCC1954, HeLa, HLF, Hs578T, HSC-3, HSC-3-M3, JIMT-1, JHH-5, JHH-6, JHH-7, Li-7, LuM1, MDA-MB-435S, MKN-74, NCI-H1650, NCI-H1975, NCI-H1993, NCI-H23, NM11, SK-OV-3, Panc-1, PLC/PRF/5, RBE, WiDr	36
**Sphere**	3T3-L1, BT474, Capan-2, DLD-1, HCT116, Hep3B, HT29, huH-1, MCF7, NCI-H226, NCI-H441, T47-D, U251	13
**Grape-like aggregation (GLA)**	HLE, MDA-MB-231, MIA PaCa-2, MKN-45, PC-3, RKO, SK-BR-3, ZR-75-30	8
**Other type of aggregation**	ACHN, FaDu (L.C.), HepG2, JHH-2, LNCaP, SUIT-2, SW480, SW620	8
**Monolayer sheet**	RT7, RWPE1, FaDu (H.C.)	3
**-**	Total number of cell lines tested	67

L.C., low concentration. H.C., high concentration.

Among the eight cell lines that formed GLA, seven have been shown to be poorly differentiated, six of the lines were derived from adenocarcinoma, and two cell lines (PC-3 and MIA PaCa-2) possessed neuroendocrine tumor phenotypes [77, 78]. The cells with GLA phenotypes appeared to contain less areas of intercellular adhesion and more intercellular space and enlarged via the proliferation of cells and fusion of the aggregations with complicated grape-like shapes compared with the other phenotypes (Fig 1A). FaDu cells formed multiple types of aggregations including spheroids at low concentration of cells (Fig 1C), and monolayer sheets at higher concentration, 2 to 5 days post-seeding period. RT7 and RWPE1, which were established by immortalizing normal epithelial cells, formed epithelial sheets (Fig 1D), indicating that these cell lines have retained non-tumorigenic epithelial phenotypes that are close to normal epithelial cells.

It was thus suggested that while most tumor cells can form spheres/spheroids in 3D cell culture conditions, GLA of cells is indicative of another distinctive phenotype of tumor cells growing in the 3D condition.

### Difference in tumorigenicity and metastatic potentials between spheroid-forming and GLA-forming adenocarcinomas

**Fig 2:**
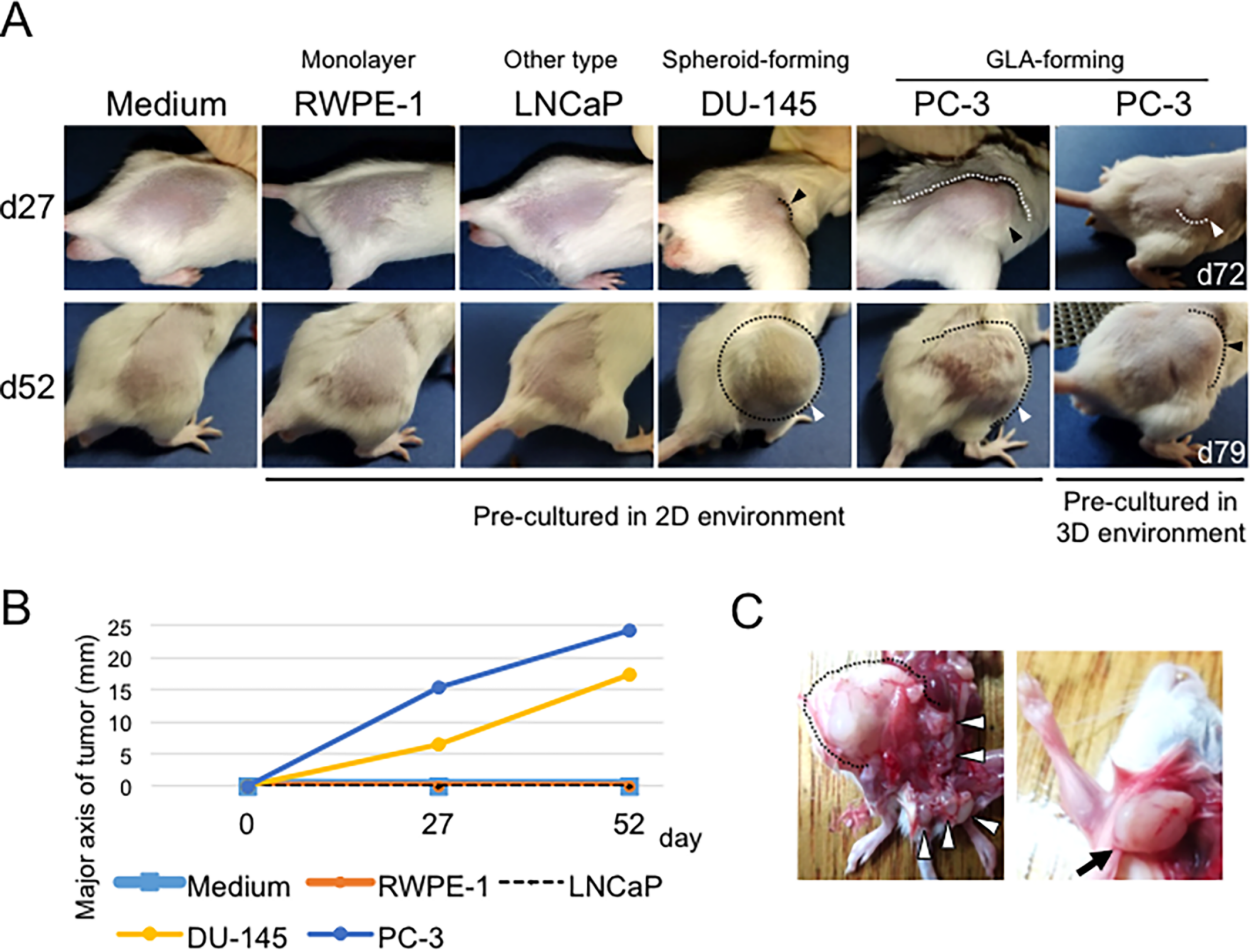
The difference in tumorigenicity and metastatic potentials between spheroid-forming and GLA-forming adenocarcinomas. (A) In vivo tumorigenicity of PC-3 (GLA-forming), DU-145 (spheroid-forming), LNCaP (other type of aggregates), and RWPE-1 (monolayer sheet-forming) cells. Each cell line was pre-cultured under the 2D conditions and transplanted to SCID mice. The PC-3 cells were pre-cultured in 2D or 3D conditions. The photographs on day 27, 52, 72, and 79 after the transplantation were shown. Arrows indicate tumors. Margins of tumors were traced with dotted lines. Tumor sizes were shown in Table 2. (B) Tumor growth of each cell line. (C) Primary tumor and lymph node metastasis of the PC-3 cells on day 52. The primary tumor was shown with dotted line. Metastasis was seen in the regional lymph nodes of the prostate (white arrows) and an axilla lymph node (arrow).

We next examined in vivo tumorigenicity of four different types of cell lines, including normal prostate epithelial cell line RWPE-1, cell aggregate-forming cell line LNCaP, spheroid-forming prostate adenocarcinoma cell lines DU-145, GLA-forming neuroendocrine adenocarcinoma cell line PC-3 (S1 Table). The medium control, RWPE-1, and LNCaP cells did not form in vivo tumor until day 52 after the transplantation, whereas DU-145 cells formed tumors with sphere morphology and PC-3 cells formed tumors at day 27 with asymmetric morphology (Fig 2A, Table 2). These morphologies of PC-3 and DU-145 tumors were consistent with their morphologies within cell aggregates formed under NCP condition in vitro (Table 1, Fig 1, later figures). These data suggest that cell aggregates formed on NCP might have similar characteristics to those found in tumors in vivo. The tumors formed by the prostate neuroendocrine cancer cell line PC-3 developed rapidly as compared to that formed by the prostate cancer cell line DU-145 (Fig 2B, Table 2). We next examined GLA-forming PC-3 cells, by pre-culturing the cells on 3D NCP before injection into recipient mice. The 3D-pre-cultured PC-3 cells formed tumors in vivo (Fig 2A). In addition, such cells metastasized to multiple lymph nodes, including regional lymph nodes of the prostate and an axilla lymph node, two months after transplantation (Fig 2C), whereas other cell lines did not metastasize to lymph nodes in this timeframe (Table 2).

**Table 2:** Tumor morphologies and metastatic potentials of the prostate-derived cell lines.

Transplant	Morphology in NCP- based 3D culture	Morphology of Tumors in vivo	Major axis (mm)		Metastasis
			d27	d52	
**Medium**	n.d.	n.d.	0	0	n.d.
**RWPE-1**	Monolayer sheet	n.d.	0	0	n.d.
**LNCaP**	Asymmetric GLA	n.d.	0	0	n.d.
**DU-145**	Spheroid	Spheroid	6.4	17.5	n.d.
**PC-3**	Asymmetric GLA	Asymmetric tumor	15.4	24.2	LN

n.d., not detected. LN, lymph node. GLA, grape-like aggregation.

These findings indicate that cell aggregates formed in the 3D environment in vitro share some properties with tumor xenografts and the GLA-forming neuroendocrine adenocarcinoma cells have an enhanced potential to develop in vivo tumors and metastasize when injected into mice.

### Quantification of size and hypoxia level of GLAs

**Fig 3:**
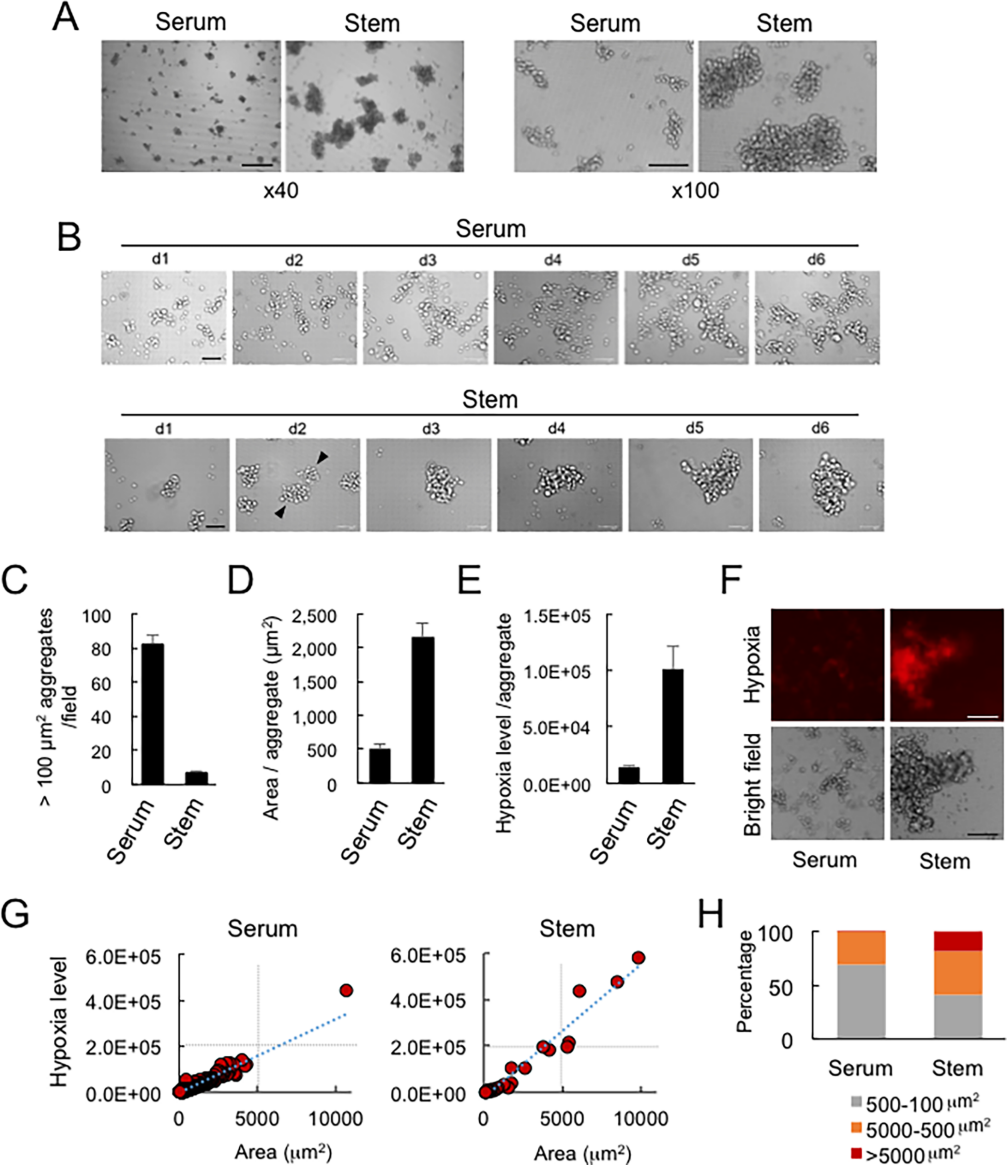
Quantification of size and hypoxia level of GLAs in serum-stimulated and stemness-induction conditions. (A) The grape-like aggregation (GLA) of PC-3 cells in the serum-contained or stem-cell medium. Scale bar, 100 μm. (B) Time-lapse imaging of maturation of GLA of PC-3 cells. Fusion of small GLAs was seen to form larger GLAs in the stem cell induction condition. Arrowheads indicate pre-fusion aggregates. Scale bar, 100 μm. (C) Numbers of cellular aggregations per field. A cellular aggregation sized more than 100 μm^2^ diameters was defined as a GLA. A well of a 96-well plate was sectionized to 9 fields, and the area of 1 field is 2,632 μm^2^. n = 4. Biological replicates. (D) The average area of GLAs. n = 3. Biological replicates. (E) Hypoxia levels of the aggregations. n = 3. Biological replicates. (F) Hypoxia imaging by using a hypoxia probe. Scale bars, 200 μm. (G) Scatter plot analysis of areas and hypoxic levels of the GLAs. GLAs in random 4 fields were analyzed. Approximate straight lines were shown. Left, n = 1338. Right, n = 27. (H) The difference in sizes of GLAs. Gray, 100 to 500 μm^2^; orange, 500 to 5000 μm^2^. Red, > 5000 μm^2^.

There is no standardized definition of cellular aggregation. In the present study, we first defined a cellular mass larger than 100 μm^2^ as a cellular aggregation for the purpose of these studies and quantified the sizes and hypoxia levels of such structures. Moreover, as the GLA phenotype has not yet been thoroughly investigated, we tested several tissue culture conditions. We cultured PC-3 cells on NCPs in serum (BSA)-containing medium and in mTeSR1, stem-cell-inducing medium. Smaller GLA (sGLA) were seen in the serum-containing medium, whereas larger GLA (lGLA) occurred in the stem cell medium (Fig 3A). PC-3 cells with axonal projections were also observed under the serum-containing 2D conditions (an arrow), indicating serum stimulation of neuroendocrine differentiation.

Next, we examined GLA formation in PC-3 cells growing for 6 days on NCPs. The PC-3 cells formed GLAs which showed collective cell migration and fusion (Fig 3B). PC-3 cells proliferated in serum-containing medium on the NCP, and the number of GLAs was increased (Fig 3B, upper photomicrographs), whereas the PC-3 cell aggregates appeared more likely to fuse to each other (arrowheads), forming larger GLAs in the stem-cell medium (Fig 3B, lower photomicrographs). The PC-3 cells within GLAs showed morphological heterogeneity, including small cells and large cells when cultured in serum-containing medium, but showed relatively greater homogeneity, consisting largely of small cells in the stem-cell medium.

To further characterize the GLA phenotype of PC-3, we analyzed numbers and the average area of cellular aggregations in a high content screening system. The average number of cellular aggregations formed in the serum-containing medium was 82.2, whereas that in the mTeSR1 was 6.8 (Fig 3C). The average area of the cellular aggregations formed in serum-containing medium was 515.1 μm^2^, whereas that in the mTeSR1 was 2090.1 μm^2^ (4.1-fold higher than that in the serum-containing medium) (Fig 3D).

Next, we examined hypoxia levels in the cellular aggregations. The average hypoxia level (= fluorescence intensities) in the cellular aggregations formed in the serum-containing medium was 14135.5, whereas that in the mTeSR1 was 97611.8 (6.9-fold higher than that in the serum-containing condition) (Fig 3E and 3F). Subsequently, we examined hypoxia levels and areas of single cellular aggregations by scatter plot analysis. Larger hypoxic cellular aggregations were formed in the stem-cell medium as compared to the serum-containing condition (Fig 3F, 3G and 3H). The 99.93% of cellular aggregations was sized less than 5,000 μm^2^ with hypoxia levels less than 150k and 0.07% of cellular aggregations was sized > 5,000 μm^2^ in the serum-containing medium. In contrast, 18.52% of cellular aggregations was >5,000 μm^2^ and 91.48% of cellular aggregations was sized between 100 to 5,000 μm^2^ (Fig 3H).

Therefore, factors contained in serum appear to promote the formation of small aggregations of cells along with proliferation but appear to decrease fusion of aggregations, whereas the stem-cell medium promoted enlargement of cellular aggregations.

### Enlargement of GLA due to slow cell proliferation and inter-GLA fusion in the 3D stemness-inducing nanoenvironment

**Fig 4:**
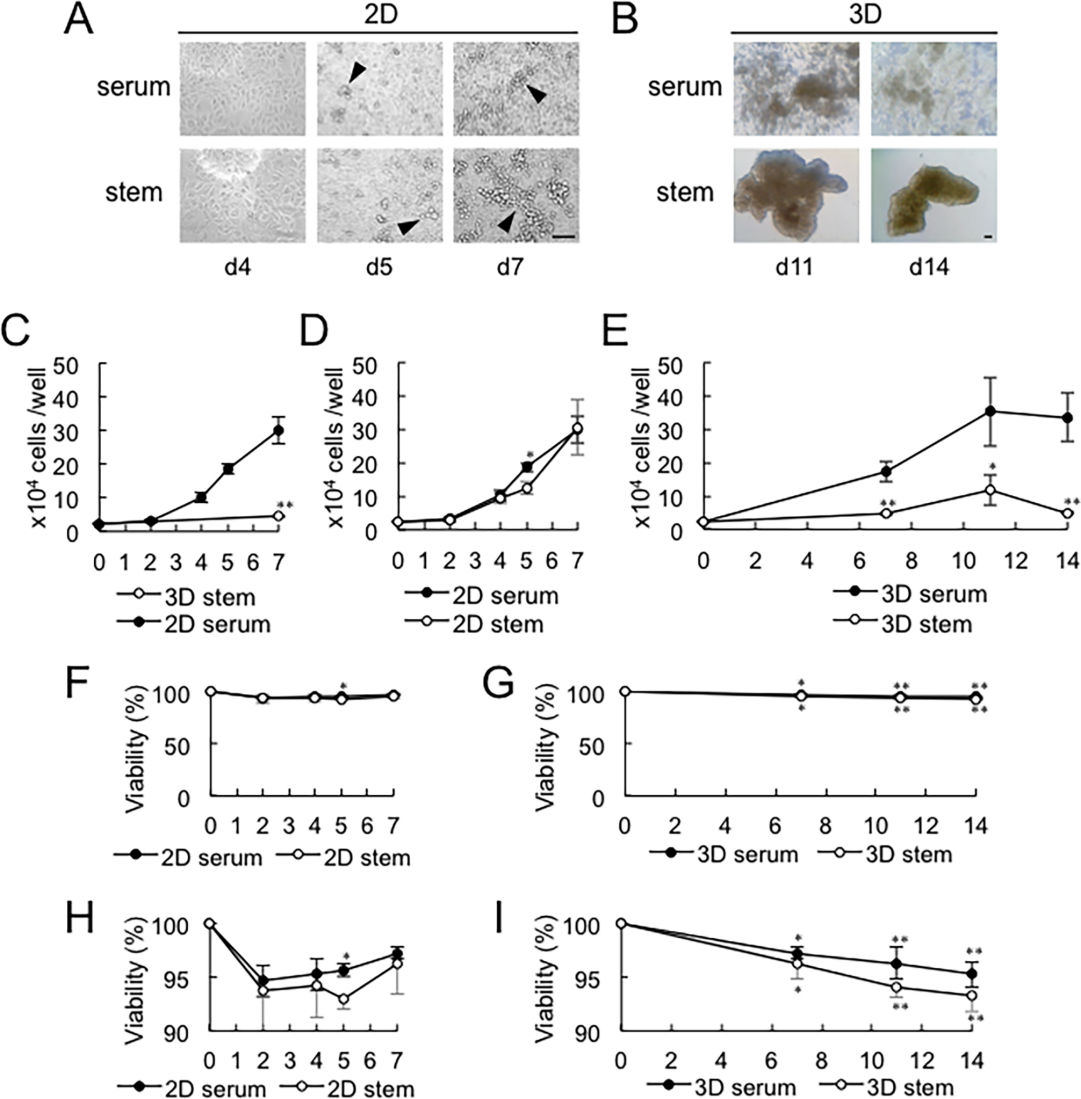
Enlargement of GLA due to slow cell proliferation and inter-gla fusion in the 3d stemness-inducing nanoenvironment. (A) Representative photomicrographs of PC-3 cells after reaching confluent. Cells were cultured in the 2D condition. Cellular morphologies at day 4, 5, and 7 were shown. Arrowheads indicate GLA on the 2D monolayer cells. Scale bar, 100 μm. (B) Representative photomicrographs of PC-3 cells in the 3D culture condition. Cellular morphologies at day 11 and 14 were shown. Scale bar, 100 μm. (C) Growth curves of PC-3 cells cultured in 2D serum-contained and 3D stem cell medium conditions. Cells were cultured in a 96-well plate. **P < 0.01 (2D serum vs 3D stem), n = 3. (D) Growth curves of PC-3 cells cultured in 2D serum-contained and 2D stem cell medium conditions. *P < 0.05 (2D stem vs 2D serum), n = 3. (E) Growth curves of PC-3 cells cultured in 3D serum-contained and 3D stem cell medium conditions. *P < 0.05 (3D stem vs 3D serum), n = 3. **P < 0.01 (3D stem vs 3D serum), n = 3. (F-H) Viabilities of PC-3 cells cultured in 2D or 3D conditions in serum-contained or stem cell media. Same data with different vertical axis values were shown between F and H and between G and I. (F, H) P < 0.05 (2D serum vs 2D stem), n = 3. (G, I) *P < 0.05 (vs day 0), n = 3. **P < 0.01 (vs day 0), n = 3. 3D serum d0 vs d7, P = 0.028. 3D serum d0 vs d11, P = 0.0052. 3D serum d0 vs d14, P = 0.0012. 3D stem d0 vs d7, P = 0.0138. 3D stem d0 vs d11, P = 0.0007. 3D stem d0 vs d14, P = 0.0004.

CSCs have been shown to proliferate relatively gradually with a long cell cycle and slow metabolism, and often enter into a status of dormancy [52]. We hypothesized that 3D microenvironment could induce cancer cells to de-differentiate into slowly cycling stem cells, in addition to promoting the aggregation morphology and stem cell-associated gene expression profile. We found that 3D GLAs of PC-3 cells were increased on the confluent cellular sheets even in 2D culture condition, suggesting the strong GLA-forming ability of this adenocarcinoma cell line (Fig 4A). Larger GLAs but with reduced frequency were formed via inter-GLA fusion in the 3D stem environment, whereas relatively smaller sized GLAs but with increased number were formed in the 3D serum-contained condition (Fig 4B). (Quantitative analysis of size and number of cell aggregates were shown in Fig 3).

We next measured the rate of cell proliferation and viability of the adenocarcinoma cells in these different conditions. The PC-3 cells grew in the 2D conditions with a doubling time of 1.1 to 2.0 days even after reaching confluency, whereas the cells grew slowly in the 3D stem condition with a doubling time of 7.2 days (Fig 4C). The proliferation of PC-3 cells under the 2D environment did not show a large difference between the conditions of serum-stimulation and stem cell medium (doubling time of 1.9 days and 1.9 days, respectively) (Fig 4D). We next examined the growth of PC-3 cells in the 3D condition for 2 weeks. Serum promoted cell growth even in the 3D condition with a doubling time of 2.4 days, whereas the 3D stem cell-inducing condition caused cells to grow very slowly with a doubling time of 7.2 days (Fig 4E, Table 3).

**Table 3:** Doubling time of the neuroendocrine PC-3 cells in the 4 different conditions.

Condition	2D serum	2D stem	3D serum	3D stem
**Doubling time**	1.9 days	1.9 days	2.4 days	7.2 days

In the 3D culture condition, the number of cells was decreased between day 11 and day 14 (Fig 4E), suggesting that cells died in this period. Viabilities of aggregate-forming PC-3 cells were as usual in these four different conditions (Fig 4F and 4G), while there were tendencies that cellular viabilities were higher in the serum-contained conditions as compared to the stem cell medium conditions (Fig 4H and 4I) and viability was decreased depending on the cell culture period in the 3D condition (Fig 4I).

These findings suggested that the 3D and stemness-inducing conditions cause the adenocarcinoma cells to enter into slow cycling stemness phase, whereas 2D culture condition and serum stimulate the cancer cells to grow rapidly.

### Stem-cell-inducing medium promotes intercellular adhesion whereas serum stimulation promotes morphological diversity of the neuroendocrine adenocarcinoma cells

**Fig 5:**
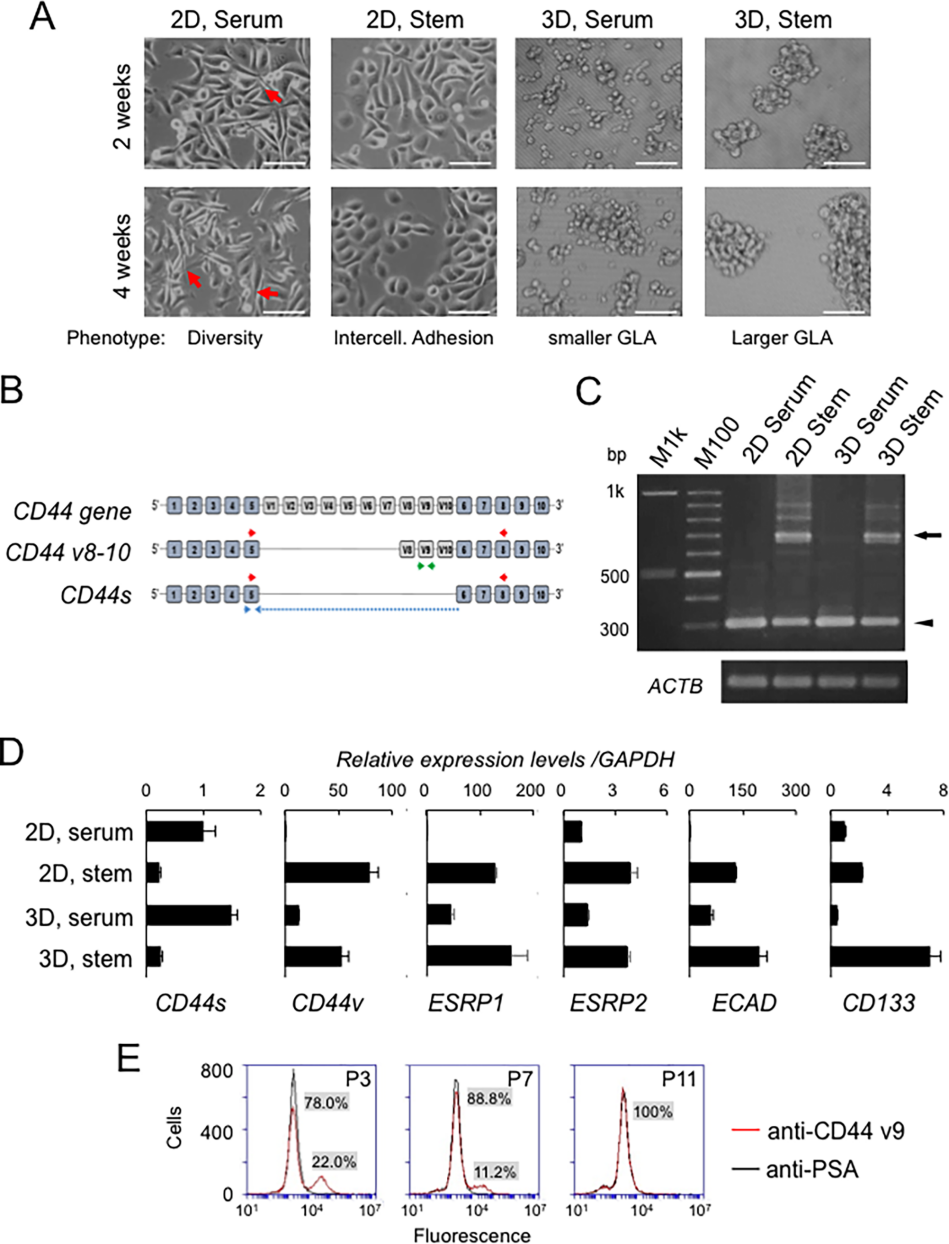
Gene expression switching of Epithelial-Splicing Regulatory Proteins (ESRPs), CD44 variant, and stem cell markers depending on cell culture nanoEnvironments. (A) Representative morphologies of PC-3 cells cultured in the 4 different conditions. Cells were cultured in 10% serum-containing F12K medium or mTeSR1 stem-cell medium on 2D plates or 3D NCPs. Arrows indicate projections of cells. Scale bars, 100 μm. (B) Schematic structures of *CD44* gene, *CD44 variant 8–10* (*CD44v8-10*) and *CD44 standard* (*CD44s*). Blue and gray rectangles represent standard exons (exon 1 to 10) and variant exons (V1 to V10), respectively. The red primer pair is for all variants and the CD44s. The green primer pair is for CD44v containing exon V9. The blue primer pair is for CD44s only. (C) Agarose gel electrophoresis analysis of RT-PCR amplicons of CD44v and CD44s. An arrow indicates *CD44v8-10* amplicon. An arrowhead indicates *CD44s* amplicon. M1k, a 1 kbp DNA ladder marker. M100, a 100 bp DNA ladder marker. *ACTB*, β-actin mRNA as an internal control. (D) qRT-PCR analysis of stem-cell-related and epithelial-splicing regulatory genes. The mRNA expression levels of *CD44s*, *CD44v*, *ECAD/CDH1*, *ESRP1*, *ESRP2*, and *CD133* were examined. Relative mRNA expression levels versus those of *GAPDH* are shown. n = 3. (E) Flow cytometry analysis of CD44v9. PC-3 cells were cultured in serum-containing medium and passage number 1, 7, and 13 were examined by flow cytometry. An anti-prostate-specific antigen (PSA) antibody was used as a negative control. Serum promoted differentiation of the cells and reduced stemness.

Next, we examined further morphological changes of the PC-3 cells grown in 2D and 3D conditions. The PC-3 cells showed evidence of morphological diversity (heterogeneity) including spindle-shaped cells, neuronal cells with axonal projections (arrows), intercellularly adhesive cells, and round-shaped cells in the serum-containing medium in 2D condition (Fig 5A). In contrast, PC-3 cells appeared to possess increased numbers of intercellular adhesions in the stem-cell medium in 2D condition. The PC-3 cells formed smaller cellular aggregations in serum-containing 3D condition and formed larger cellular aggregations with more intercellular adhesion in the stem-cell medium in 3D condition. These results indicate that the intercellular adhesion and cellular aggregation are strengthened in the stem-cell medium both in 2D and 3D conditions, whereas the loss of intercellular adhesion and cellular differentiation are promoted in the serum-containing medium.

### Gene expression switching of Epithelial-Splicing Regulatory Proteins (ESRPs), CD44 variant, CD133, and E-cadherin depending on cell culture nanoenvironments.

We next examined expression of *CD44v*, *CD44s*, *ESRP1*, *ESRP2*, *ECAD/CDH1*, *and CD133/PROM1* in the cells with epithelial intercellular adhesion induced by the stem-cell medium (2D stem and 3D stem) and the cells with EMT induced by serum stimulation (2D serum and 3D serum), whose relative morphologies were shown above. The PCR amplicon sized approx. 700 bp was consistent with *CD44v8-10*, which was expressed in the 2D and 3D stemness condition (Fig 5B and 5C). Minor PCR amplicons sized approx. 680 bp, 900 bp, and 800 bp that were consistent with *CD44v6-10* were also detected in the stemness conditions; however, neither of the amplicons consistent with *CD44v2-10* (should be approx. 1100 bp) nor *CD44v9* (should be approx. 600 bp) were detected. The PCR amplicon sized approx. 304 bp was consistent with the CD44s, which was expressed in the 2D and 3D serum-containing conditions at high levels, but at lower levels in the 2D and 3D stemness conditions. We next examined the mRNA levels of the v9 region-containing CD44v using the primer pairs as shown in Fig 5B (green) and of CD44s using the CD44s-specific primer pair as shown in Fig 5B (blue). The *CD44s* mRNA was expressed at high levels in the 2D and 3D serum-containing conditions, but at relatively low levels in the stem-cell medium, further indicating that *CD44s* was expressed under the EMT-promoting conditions. *CD44v* containing the v9 region was expressed at high levels in the 2D and 3D stem-cell conditions (Fig 5D), whereas this transcript was at low levels in the serum-containing condition, consistent with the agarose gel electrophoresis analysis as shown in Fig 5C. These results indicated that *CD44v8-10* is expressed in the stem-cell-induced adhesive PC-3 cells, while CD44s is expressed at high levels in the serum-containing conditions. The mRNA levels of *ECAD/CDH1*, *ESRP1* and *ESRP2* were increased in the stemness conditions, with intercellular adhesion and 3D conditions (Fig 5D) but were at a low level in the serum-containing 2D condition, coincidently with the expression pattern of *CD44v8-10* and with the cell/cellular aggregation morphologies as shown in Fig 3A. We next examined *CD133/PROM1* expression, which has been known as a prostate CSC marker as well as tissue stem-cell marker (54). CD133 was expressed at a high level in the 3D stem cell aggregations and 2D stem-cell conditions as compared to those in the serum-containing conditions.

We next examined the cell-surface expression of CD44 variant containing V9 region at the protein level. The CD44v containing the V9 region was presented on approx. 48% of PC-3 cells at passage number 2 (S1 Fig); however, it was lost by culturing the cells in serum-containing medium (22.0% at P3, 11.2% at P7, and 0% at P11) (Fig 5E). Some factors contained in serum might promote differentiation of the cells and reduced stemness.

These results indicate that *CD44v8-10*, *ESRP1*, *ESRP2*, *ECAD*, and *CD133* are expressed at relatively high levels in the stem-cell condition in PC-3 cells with intercellular adhesion, whereas *CD44s* is expressed at high levels in the serum-induced differentiated cells.

### Gene expression profiling of CSC markers in the 2D and 3D culture nanoenvironments

**Fig 6:**
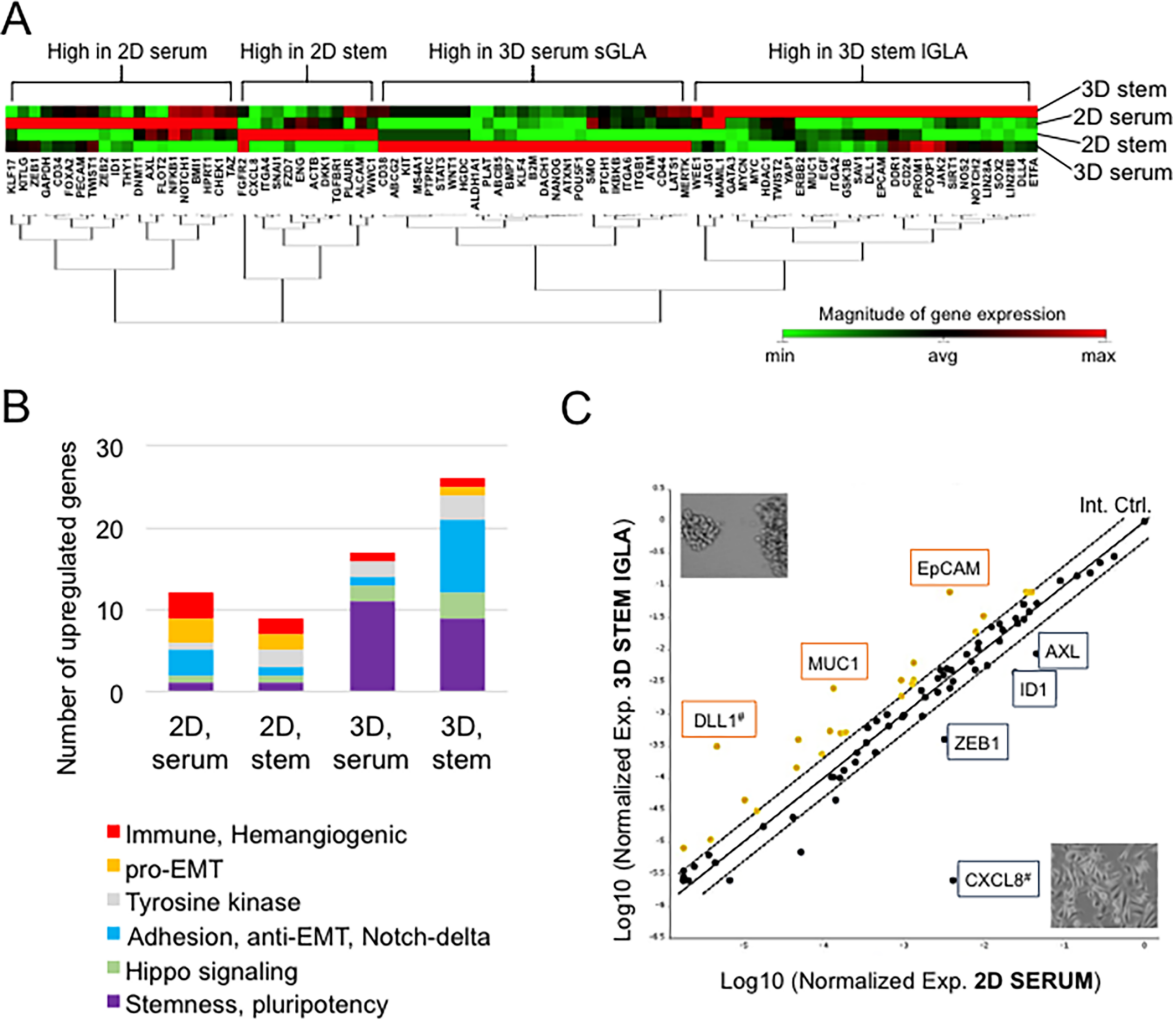
Gene expression profiling of CSC markers in the 2D and 3D culture nanoenvironments. PC-3 cells were cultured in four different conditions designated: 2D, serum; 2D, stem; 3D, serum; 3D, stem conditions. (A) Clustergram and dendrogram analysis of stem-cell-related genes. (B) Building block analysis of stem-cell-related genes. (C) Scatter plot analysis. Genes were plotted according to the mRNA expression levels in the 3D stem-cell condition (ordinate) and the 2D serum-containing condition (abscissa). ^#^These gene’s average threshold cycle is relatively high (> 30) in any sample, and is reasonably low in the other sample (< 30).

To connect the altered aggregation phenotypes with gene expression signatures, we carried out gene expression profiling of CSC markers under 2D and 3D conditions. We tested four conditions- 3D culture in the stem-cell medium on the NCPs (3D stem), 3D culture in serum-containing medium on the NCPs (3D serum), 2D culture in mTeSR1 medium (2D stem), and 2D culture in serum-containing medium (2D serum). The gene expression signatures were analyzed in clustergram (Fig 6A). The number of genes expressed at high level in the 3D, stem-cell condition was 29 in total, including *DLL1*, *MUC1*, and *EPCAM*. The number of genes expressed at high level in the 3D, serum-containing condition was 30 genes in all, including *DACH1*, *SCA1/ATXN1*, *ALDH1A1*, and *NANOG*. Twelve genes were expressed at high level in the 2D, stem-cell conditions, including *CXCL8*. The Number of genes expressed at high level in the 2D, serum-containing condition was 19, including *ZEB1*, *AXL*, and *ID1*.

Next, in order to look down upon the different gene expression signatures, we categorized the genes into 6 groups, including: a group of transcripts promoting stem cell properties and pluripotency; a group of hippo signaling; a group of transcripts promoting intercellular adhesion, anti-EMT and Notch signaling; a group of tyrosine kinase genes; a pro-EMT group; and a group of immunity and hemangiogenic genes. According to these categories, we performed building block analysis (Fig 6B). Stemness- and pluripotency-related genes were expressed in 3D conditions at high levels. Nine stem cell marker genes were expressed in the 3D stem-cell condition (*SOX2*, *MYC*, *MYCN*, *GATA3*, *LIN28A*, *LIN28B*, *JAK2*, *CD44*, *PROM1/CD133*), 11 stem cell marker genes were expressed in the 3D serum-containing condition (*OCT4/POU5F1*, *NANOG*, *KLF4*, *ALDH1A1*, *WNT1*, *STAT3*, *JAK2*, *CD44*, *PROM1/CD133*, *BMP7*, *SCA1/ATXN1*), whereas only one stem cell marker gene was expressed in the 2D conditions (*FZD7*, *BMI1*) (purple blocks), indicating that stemness-related genes were upregulated in the 3D NCP condition and could be involved in the formation of tumor cell aggregations.

Nine intercellular adhesion genes including anti-EMT genes and Notch/Delta/Jag signal-related genes (*EPCAM*, *ALCAM*, *CD24*, *NOTCH1*, *NOTCH2*, *DLL1*, *DLL4*, *JAG1*) were expressed in the 3D stem-cell condition, whereas less than four adhesion genes (*DACH1*, *TGFBR1*, *SNAI1*, *ZEB1*, *ZEB2*, *TWIST1*) were expressed in the other condition tested, indicating that adhesion-related, anti-EMT, and Notch signaling genes were upregulated in the 3D stem-cell condition and contributed to the formation of large aggregations.

Three Hippo signaling genes were expressed in the 3D stem-cell condition (*YAP1*, *LATS1*, and *SAV1*), 2 Hippo signaling genes were expressed in the 3D serum-containing condition (*LATS* and *MERTK*), whereas 1 Hippo signaling genes were expressed in the 2D conditions (*WWC1*, *TAZ*), indicating that Hippo signaling could promote cell aggregation.

Three immune and hemangiogenic genes (*CD34*, *ID1*, *CD31/PECAM*) and 3 pro-EMT genes were expressed in the 2D serum-containing condition, whereas less than 2 immune and hemangiogenic genes (*CXCL8*, *ALCAM*), or pro EMT genes were expressed in the 3D conditions, indicating that serum could promote cell differentiation and heterogeneity in the 2D culture condition. These results indicate that stem-cell gene expression signature accurately represents the molecular background of the cell morphology including the size of cellular aggregations and diversity/heterogeneity.

### The NCP-based 3D environment enables neuroendocrine adenocarcinoma cells to express stem cell marker genes and oncogenes

**Fig 7:**
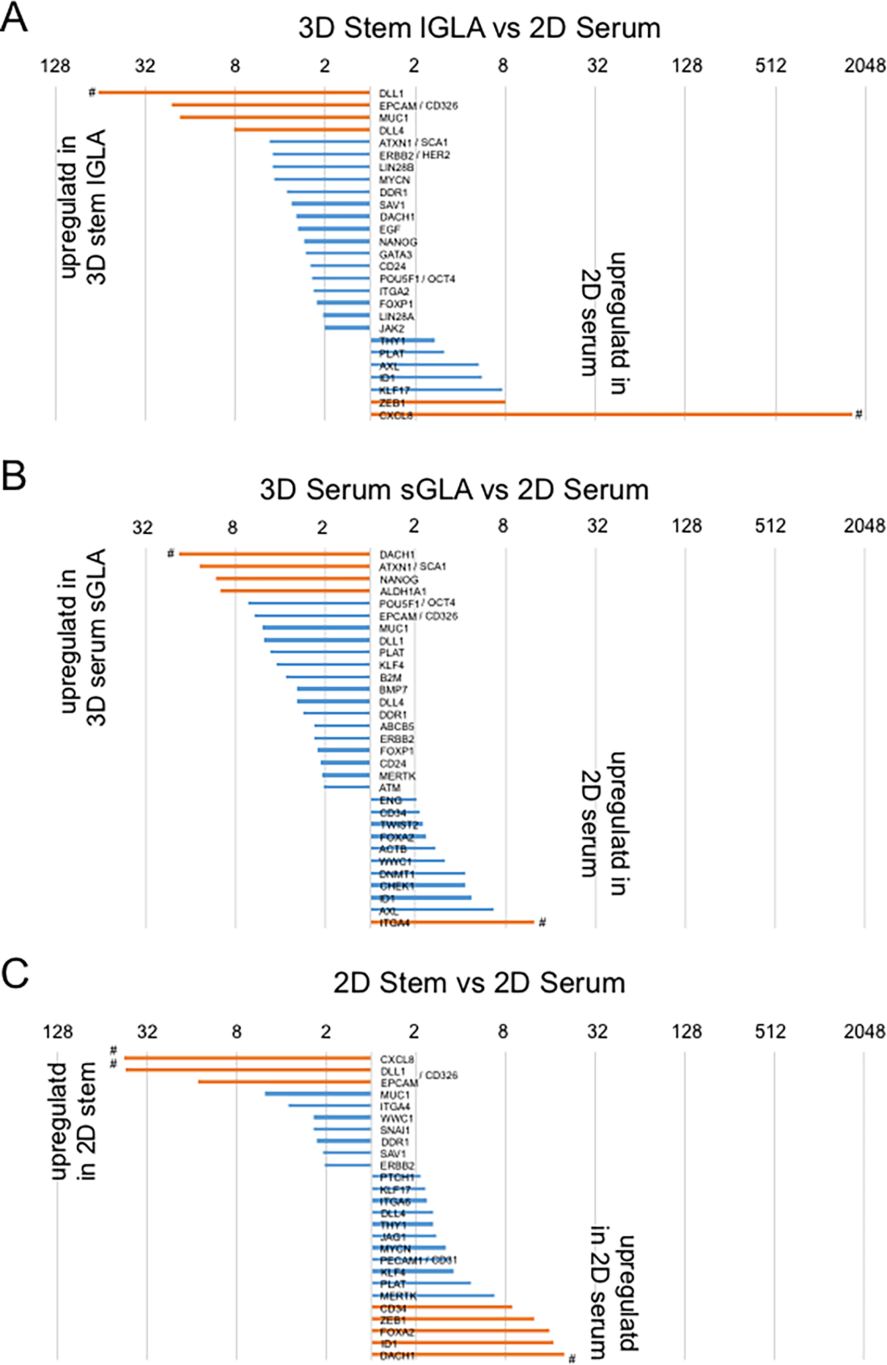
Gene expression of stem cell markers in the 3D GLAs and differentiation markers in the 2D cells. (A) Fold expression changes in the large GLA-forming 3D stem-cell condition vs 2D serum-containing condition. (B) Fold expression changes in the small-GLA-forming 3D serum-containing condition vs 2D serum-containing condition. (C) Fold expression changes in the 2D stem-cell condition vs 2D serum-containing condition. Orange bars, gene expression changed more than 8-fold. Blue bars, gene expression changed more than 2-fold and less than 8-fold. ^#^These gene’s average threshold cycle is relatively high (> 30) in any sample, and is reasonably low in the other sample (< 30).

We next examined fold changes of gene expression in the four conditions of PC-3 cells by scatter plot analysis (Fig 6C), bar graphs (Fig 7A–7C) and ranking (Tables 4 and 5). Gene expression of *DLL* (65.8-fold), *EPCAM* (21.3-fold), and *MUC1* (18.8-fold) were induced in the 3D stem-cell condition as compared to the 2D serum-containing condition (Figs 6C and 7A, Table 4). The gene expression of *DLL*, *EPCAM*, and *MUC1* were also at higher levels in the 2D stem-cell and 3D serum-containing conditions as compared to the 2D serum-containing conditions (Fig 7C). Expression of hemangiogenic genes *CXCL8* (1629.3-fold) and *ID1* (5.6-fold) and EMT-related genes *ZEB1* (8.0-fold) and *KLF17* (7.6-fold), and RTK gene *AXL* (5.2-fold) were at high levels in the 2D serum-containing condition as compared to the 3D stem-cell condition (Figs 6C and 7A, Table 5). Expression of stem cell marker genes including *SCA1/ATXN1* (13.8-fold), *NANOG* (10.8-fold), *ALDH1A1* (10.1-fold), *OCT4/POU5F1* (6.5-fold), *KLF4* (4.2-fold) and of CSC marker genes including *EPCAM* (5.9-fold) and *MUC1* (5.3-fold) increased in the 3D serum-containing condition as compared to the 2D serum-containing condition, indicating that the 3D aggregation of cells on NCPs induces stemness. These results indicate that pluripotent stemness genes and CSC genes involve cellular aggregation on NCPs.

**Table 4:** A gene list upregulated in cell aggregation (3D stem / 2D serum).

Gene symbol	Fold change	Function	Reported as oncogene?	Ref
**DLL1**	65.8^#^	Delta-like ligand of Notch. Adhesion.	Controversial	[79]
**CD326/** **EpCAM /ESA**	21.3	EpICD forms complex with FHL2/β-catenin/TCF to activate myc and cyclin genes. Form complex with tetraspanins, claudin 7, CD9, and CD44, and found in exosome.	Oncogenic	[56, 80, 81]
**MUC1**	18.8	Encodes mucin-1 Oncoglycoprotein. Extracellular MUC1-N and membrane-bound MUC1-C have distinctive roles. MUC1-C forms complexes with EGFR and RTKs via Galectin 3 and activate AKT-mTOR pathway. MUC1-C dimerized and was transported to nuclei and associate with STAT1/3, NF-kB RelA, ERα, or TCF/catenin, or to the mitochondrial outer membrane where it blocks apoptosis. Found in CSCs and exosomes. Immunosuppressive.	Oncoprotein	[82–85]
**DLL4**	8.2^#^	Delta-like ligand of Notch. Adhesion. A target of HIF2α. Tumor cell DLL4 binds to Notch1 on the surface of endothelial cells and contribute to extravasation and metastasis.(A)	Controversial	[79]
**ATXN1** **/SCA1**	4.7	Expresses in tumor initiating cells (TICs) or CSCs.	expressed in TIC or CSC	[23, 86, 87]
**ERBB2/** **HER2** **/NEU**	4.5	A member EGFR family. Form heterodimers with other members of EGFR/ERBB. The EGF ligand family is composed of EGFs and neuregulins (NRGs). RTK. Found in exosome.	Oncogene	[88, 89]
**LIN28B**	4.5	Stemness. Repress let-7 tumor suppressor miRNAs.	Oncogenic	[90–92]
**MYCN**	4.4	Found in neuroblastoma. Stemness. Pluripotency.	Oncogene	[93–96]
**DDR1**	3.6	Collagen receptor. RTK.	Oncogenic	[97, 98]
**SAV1**	3.4	Hippo signal.	Tumor suppressor	[99–101]
**DACH1**	3.1^#^	Anti-EMT, adhesion.	Tumor suppressor	[102–105]
**EGF**	3.1	A ligand of EGFR family.	Ligand of oncoprotein	[88, 89]

Genes expressed at high levels in the 3D stem aggregation condition as compared to the 2D serum differentiation condition were listed.

^#^These gene’s average threshold cycle is relatively high (> 30) in any sample, and is reasonably low in the other sample (< 30).

**Table 5:** A gene list upregulated in the 2D serum-stimulated differentiation condition (2D serum / 3D stem).

Gene symbol	Fold change	Function	Reported as oncogene?	Ref
**CXCL8**	1629.3^#^	Pro-angiogenic chemokine.	-	[106–108]
**ZEB1**	8.0	Pro-EMT. Induced by catenin/TCF complex.	Oncogenic	[109–112]
**KLF17**	7.6	pro- or anti-EMT. KLF17-fusion genes in myoepithelial tumors.	Controversial	[113–115]
**ID1**	5.6	Neurogenesis, angiogenesis. Induced by BMP via Smad-binding elements. Pro-metastatic TF. Biomarker of endothelial progenitors.	Oncogenic	[116, 117]
**AXL**	5.2	A member of TAM RTK. Phosphatidylserine-sensing RTK. Apoptotic cell clearance, anti-viral, blood vessel integrity. Biomarker of poor prognosis of cancer. pro-metastatic. chemoresistance.	Oncogenic	[118]
**PLAT**	3.1^#^	protease, plasminogen activator tissue type, tPA. Activating protease for metalloproteinases.	-	[119, 120]

Genes expressed at high levels in the 2D serum differentiation condition as compared to the 3D stem aggregation condition were listed.

^#^These gene’s average threshold cycle is relatively high (> 30) in any sample, and is reasonably low in the other sample (< 30).

Eight genes in the top 12 genes upregulated in the 3D stem-cell conditions have been reported to be oncogenes, oncogenic, or oncoproteins (Table 4), encoding the epithelial cell adhesion molecule EpCAM/CD326, mucin 1 (MUC1), stem cell antigen 1 also known as ataxin 1 (SCA1/ATXN1), the ERBB2/HER2/NEU RTK, LIN28B a regulator of miRNA, N-MYC, a collagen receptor tyrosine kinase DDR1, and epidermal growth factor (EGF). These results indicate that the 3D culture condition on the NCPs let the GLA of PC-3 cells express multiple oncogenes and stem-cell marker genes.

### Secretion of EpCAM-exosomes, CD9-exosomes, and HSP90 by neuroendocrine stem cell aggregates in the 3d culture nanoenvironment

**Fig 8:**
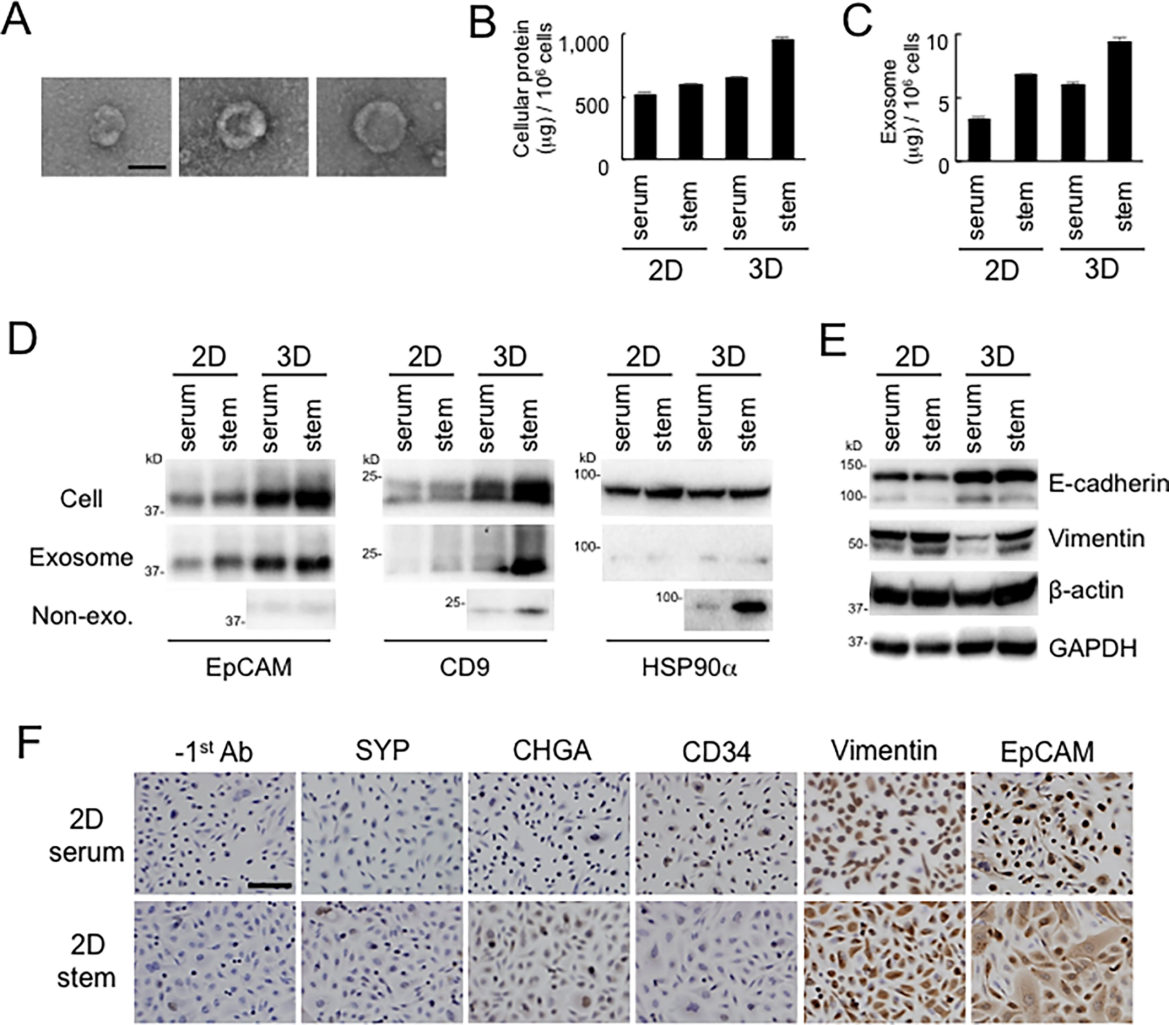
Secretion of EpCAM-exosomes, CD9-exosomes, and HSP90 by 3D aggregates of multipotent neuroendocrine adenocarcinoma cells. (A) TEM of exosomes secreted by PC-3 cells. Scale bar, 100 nm. (B, C) Cellular (B) and exosome (C) protein concentrations per million cells. PC-3 cells were pre-cultured the four different conditions and further cultured in serum-free medium for 2 days to prepare exosome and non-exosome fractions. (D) Western blotting analysis of EpCAM, CD9, and HSP90α in cellular, exosome, and non-exosome fractions. Each protein sample per 3 x 10^5^ cells was used for analysis of exosome, per 1 x 10^5^ cells was used for analysis of non-exosome fraction, and per 2 x 10^4^ cells was used for analysis of cell lysates. (E) Western blotting analysis of E-cadherin and Vimentin. Each protein sample per 2 x 10^4^ cells was loaded. β-actin and GAPDH were analyzed as loading controls. (F) Immunocytochemistry of EpCAM, vimentin, chromogranin A (CHGA), synaptophysin (SYP), and CD34 in the 2D culture conditions. Scale bar, 50 μm. Percentages of positive cells were shown in Table 4. Photomicrographs taken at a 20 x magnification is shown in S1 Fig.

It has been recently shown that various types of cells secrete exosomes which is one kind of extracellular vesicles of approx. 100 nm diameter and carry various types of nucleic acid, lipid, and proteins [41, 121–124], including tetraspanin family, EpCAM, and HSP90. EpCAM (also known as ESA or CD326) has been shown to be oncogenic and found in CSCs as well as cancer exosomes [56, 80, 81]. In the present study, the mRNA level of EpCAM was increased in the 3D stem cell medium environment (Table 4, Figs 6 and 7). HSP90 is a molecular chaperone whose expression is elevated in cellular stress and cancer cells, and consists of HSP90α, an inducible type, and HSP90β, a constitutively expressed housekeeping type [125–127] while extracellular HSP90α [128–130] and exosomal HSP90 [122, 131–133] has been recently found. Therefore, we examined whether aggregates of neuroendocrine PC-3 cells secrete EpCAM-exosomes, HSP90-exosomes and/or extracellular non-exosomal HSP90 in 3D serum-stimulated and stemness environments. Extracellular vesicles of approx. 100 nm diameter surrounded by putative lipid bilayers were found under TEM in the exosome fraction prepared from the culture supernatant of PC-3 cells (Fig 8A). We next examined protein storage and exosome secretion by PC-3 cells in 2D or 3D conditions. The protein concentration per million cells was 528.8 μg in the 2D serum-contained condition, 602.5 μg in the 2D stem cell medium condition, 656.8 μg in the 3D serum-contained condition, and 959.9 μg (1.8-fold) in the 3D stem cell medium condition (Fig 8B). Exosome secretion per million cells was 3.4 μg in the 2D serum-contained condition, 6.1 μg (1.8-fold) in the 3D serum-contained condition, 6.8 μg (2.0-fold) in the 2D stem cell medium condition, and 9.5 μg (2.8-fold) in the 3D stem cell medium condition (Fig 8C). These findings suggest that approximately 1% of cellular proteins were secreted within exosomes in 48 hours, and 3D stem cell aggregates may store more proteins in / between the cells and secrete approximately 3-fold exosomes than 2D differentiated cells.

We next examined levels of EpCAM, CD9 (tetraspanin 4), and HSP90α in cells, exosomes, and non-exosomal secretory fractions. Cellular and exosomal EpCAM levels were increased in the 3D and stem cell medium conditions as compared to those in the 2D differentiated condition (Fig 8D), indicating that EpCAM was induced in the 3D stem cell aggregates and secreted with exosomes. Cellular and exosome CD9 levels were significantly high in the 3D stem cell medium environment as compared to those in 2D or serum-contained conditions (Fig 8D), indicating that biosynthesis and secretion of exosomes increased in the 3D stem cell aggregates. Non-exosomal CD9 was also found in the culture supernatant of 3D stem cell aggregates, indicating that lipid bilayer of exosomes might be broken and released CD9. Non-exosomal HSP90α was detected abundantly in the supernatant of 3D stem cell aggregates as compared to that of the 3D serum-stimulated cells (Fig 8D).

These findings suggest that biogenesis and secretion of CD9-exosomes, EpCAM-exosomes, and non-exosomal HSP90α are promoted in the 3D stem cell aggregates of the neuroendocrine adenocarcinoma cells while these exosome-related proteins have different kinetics.

### Multipotency of CSC-like neuroendocrine adenocarcinoma cells differentiating to mesenchymal, ductal, and hemangiogenic phenotypes

PC-3 cells were originally isolated from transformed prostate adenocarcinoma and have been shown to express a neuroendocrine cancer phenotype [134–137]. We next examined the CSC exosome marker CD326/EpCAM, EMT indicators (E-cadherin and Vimentin), neuroendocrine markers (Chromogranin A and Synaptophysin) and an angiogenesis marker (CD34) expressed in the neuroendocrine adenocarcinoma cells cultured in the 2D and 3D environments. E-cadherin expression was found to be at high levels in the 3D cell aggregates as compared to those in 2D culture condition (Fig 8E). In contrast, vimentin expression was at high levels in the 2D conditions as compared to the 3D conditions. These finding indicates that 2D and 3D culture environments are determinants of protein expression levels of E-cadherin and Vimentin. (See later figure regarding subcellular localization of E-cadherin and Vimentin in cell aggregates.)

We next examined expression of EpCAM, neuroendocrine markers, EMT markers, and CD34 in the 2D culture conditions, by performing immunocytochemistry. Vimentin-positive cell rate was 100% in both serum-containing and stem cell-induced conditions (Fig 8F, Table 6, S2 Fig). CD326/EpCAM positive cell rate was 64.5% in the presence of serum and 76.8% in stem cell induction medium. We next examined two markers of neuroendocrine cancer. Synaptophysin (SYP) positive cell rate was 3.0% in the presence of serum and that in stem cell induction medium was 6.0%. Chromogranin A (CHGA) positive cell rate was 12.1% in the presence of serum whereas that in stem cell induction medium was elevated to 42.7%. These findings indicate that induction of stemness increases a neuroendocrine phenotype of the PC-3 cells. CD34 positive cell rate was 6.4% in the presence of serum whereas that in stemness induction medium was declined to 0.6%, indicating that this neuroendocrine adenocarcinoma cell line has a potential to differentiate into hemangiogenic and endothelial cells. This cell line appeared to be CD56/NCAM-negative (S2 Fig). These findings suggest that this adenocarcinoma cell line has multipotency to differentiate into mesenchymal, neuroendocrine, and hemangiogenic phenotypes from CSC-like characteristics.

**Table 6:** Positivity rates of markers of cancer-stem, neuroendocrine, mesenchymal, and endothelial cells.

Proteins	2D Serum	2D Stem	Marker of	Refs.
**CD34**	**6.4%** (32/500)	**0.6%** (3/500)	Hemangiogenic	[56, 138, 139]
**Synaptophysin**	**3.0%** (15/500)	**6.0%** (30/500)	Neuroendocrine	[134–137]
**Chromogranin A**	**12.1%** (90/743)	**42.7%** (253/600)	Neuroendocrine	[134–137]
**CD326 /EpCAM**	**64.5%** (443/687)	**76.8%** (375/488)	CSC exosomes	[56, 80, 81]
**Vimentin**	**100%**	**100%**	Malignancy, EMT	[34, 140, 141]

Positivity rates were calculated from the results of immunocytochemistry shown in Fig 8E and S2 Fig.

**Fig 9:**
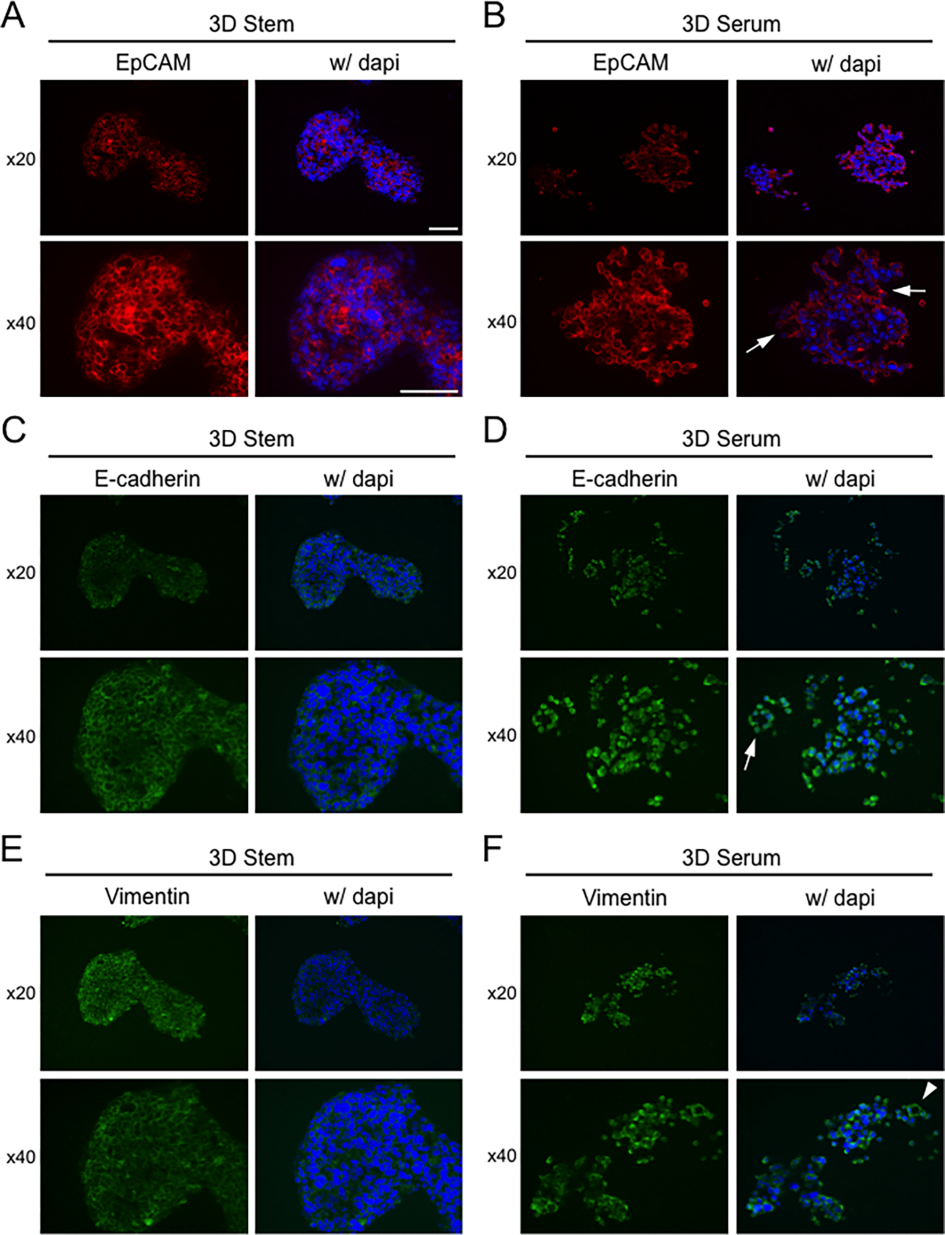
Subcellular localization of EpCAM, E-cadherin, and vimentin in the CSC-like 3D aggregates of neuroendocrine adenocarcinoma cells. PC-3 cells were cultured in 3D stem (A, C, E) and in 3D serum (B, D, F) conditions. Immunohistochemistry was carried out of EpCAM (A, B), E-cadherin (C, D) and Vimentin (E, F). DNA was stained with DAPI. Scale bars, 100 μm. Arrow indicates acinus-like structures. Arrowhead indicates duct-like structures.

It is still unknown whether cavities or ducts are formed inside stem cell aggregates of the neuroendocrine PC-3 cells. We therefore next examined morphology inside the aggregates and localization of EpCAM, E-cadherin, and Vimentin. Cell aggregates formed in the stemness-induced 3D environment were larger than those formed in the serum-contained 3D condition (Fig 9). DAPI-negative cavities or duct-like structures were found inside the cell aggregates (Fig 9). Moreover, acinus-like structures (arrow) and duct-like structures (arrowhead) surrounded by monolayer cells were found in the serum-stimulated 3D condition (Fig 9).

EpCAM localized to intercellular adhesion sites and surface of the GLAs and ductal structures (Fig 9A and 9B), indicating that EpCAM contributes to formation of the CSC-like organoids and EpCAM-exosomes are secreted and up-taken at the surface of the cells. Cavities were found inside the cell aggregates in the 3D stem environment whereas acinus-like structures (Fig 9B, arrows) were found in the cell aggregates in the 3D serum-contained differentiation condition.

E-cadherin localized to intercellular adhesion sites in the 3D stem large GLA (Fig 9C), showing mesh-like appearance. E-cadherin appeared to internalize into the cytoplasm and did not showed mesh-like appearance in the serum-stimulated GLA while an acinus-like structure (Fig 9D, arrow) was seen in the serum-stimulated differentiation condition.

Vimentin, an intermediate filament, also appeared to localize to intercellular adhesion sites in the 3D stem GLA, indicating that Vimentin lined the intercellular adhesion molecules (Fig 9E) whereas Vimentin appeared to internalize into the cytoplasm in the serum-stimulated GLA (Fig 9F). A ductal structure formed by flattened cells was seen in the serum-stimulated differentiation condition (Fig 9F, arrowhead).

Thus, EpCAM, E-cadherin and vimentin appeared to contribute to tight and robust intercellular adhesion in the organoids, in which hypoxic stem-cell properties were induced as shown in a previous figure. Although mRNA and protein levels of E-cadherin and Vimentin were altered in the 4 different conditions, subcellular localization of these proteins appeared to strongly involve organization of cell aggregates. Neither E-cadherin nor Vimentin but EpCAM appear to be responsible for intercellular adhesion for organization of the differentiating GLAs with acinus and ductal structures.

## Discussion

The development of tissue culture into a convenient methodology for basic experimentation and for development of therapeutics has made a profound impact on the rate and quality of cancer research [1]. However, for preclinical development there is always the drawback that standard tissue culture methods may not accurately model conditions found in tumors. We have compared properties of cells growing on standard tissue culture plastic plates and NCPs that permit 3D growth in vitro [5]. In addition we have compared growth stimulation by either serum containing medium or a defined growth medium concocted to favor stem cell growth. These various conditions had marked effects on the properties of prostate cancer cells. The most dramatic differences seemed to be between cells on standard dishes grown in serum containing medium and those on NCPs (Figs 1 and 3–7). The former cells grew as monolayers with a mixed morphology including both cells with epithelial and mesenchymal shapes while those on NCP formed large three-dimensional structures with intercellular adhesions that resembled the morphologies of primary tumors (Figs 2 and 5). These properties were reflected in profoundly different gene expression profiles, with monolayer, serum exposed cells expressing to high level a number of genes associate with cell differentiation (ZEB1, KLF, AXL, ID1, CXCL8) while NCP /stem cell medium cells expressing a number of oncogenes and stem cell genes (DLL1, EpCAM, Muc1, Sca1, Her2, N-Myc) (Figs 6 and 7). The NCP/stem cell medium cells also appeared to adopt a slower growing phenotype often associated with stem cells, playing roles in resistance to therapy as well as reducing stem cell exhaustion (Fig 4). In addition, the large GLA structures tended to develop hypoxic cores, again reminiscent of tumors growing in vivo (Fig 3). Our data therefore suggested that these conditions–growth on NanoCulture Plates might more effectively model the conditions found in growing prostate tumors. In addition, these cells were very efficient at secreting tumor exosomes that are thought to have important properties in cancer progression (Fig 8). EpCAM-exosomes and HSP90 were found to be abundantly secreted by the 3D stem cell aggregates of PC-3 cells. Tumor-derived exosomes were shown to transform induced pluripotent (iPS) cells into CSC-like cells, suggesting a functional role for these vesicles (51).

## Conclusion

The NCP-based 3D environment enables cells to form hypoxic stem cell aggregates and reproduce in vivo tumor status at levels of morphology and gene expression signature. These features of NCPs may be useful for advanced tumor and stem cell biology and preclinical testing of novel therapeutics.

## Supporting information

S1 Fig Flow cytometry of PC-3 cells with anti-CD44 v9 region antibodies. PC-3 cells were cultured in F12K medium containing 10% FBS. Cells were collected by using Tryple Express and incubated with 1, 3, or 7 μl of anti-CD44 v9 region PE-conjugated (red) for 10 min. An anti-PSA antibody was used as a negative control.
(PDF)

S2 Fig Immunocytochemistry of CD326/EpCAM, vimentin, CD34, chromogranin A (CHGA), synaptophysin (SYP), and CD56/NCAM in PC-3 cells in the 2D culture conditions. Percentages of positive cells were shown in Table 4. Photomicrographs were taken at a 20xmagnification.
(PDF)

S1 Table List of features of cell lines used for in vivo transplantation. (XLSX)

S2 Table List of primers. Sequences of primers to detect and quantify cDNA levels of CD44 standard, CD44 variants, ESRP1/2, E-cadherin, CD133/PROM1, GAPDH, and β-actin were listed.
(DOCX)

## Abbreviations

CSC: cancer stem cell
EMT: epithelial-to-mesenchymal transition
EpCAM: epithelial cell adhesion molecule
ESRP: epithelial splicing regulatory protein
GLA: grape-like aggregation
HSP: heat shock protein
NCP: NanoCulture Plate
NEPC: neuroendocrine prostate cancer
