# Supplementary figures and images for: Organoids with cancer stem cell-like properties secrete exosomes and HSP90 in a 3D nanoenvironment
Source: PLoS One. 2018 Feb 7;13(2):e0191109. doi: 10.1371/journal.pone.0191109 (PMC5802492; doi:10.1371/journal.pone.0191109)

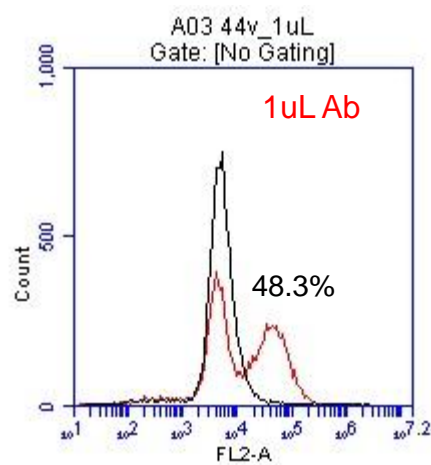

P2

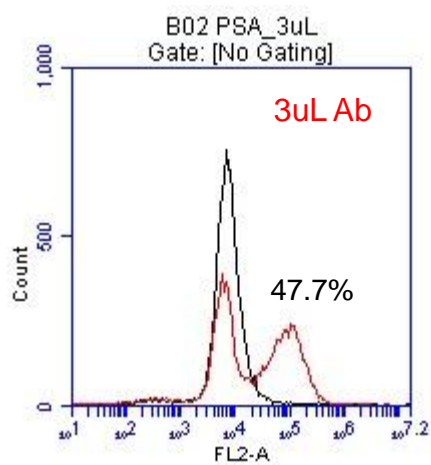

P2

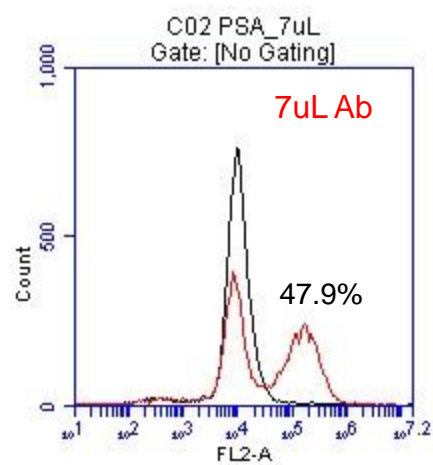

P2

Supplement: S1 Fig — PC-3 cells were cultured in F12K medium containing 10% FBS. Cells were collected by using Tryple Express and incubated with 1, 3, or 7 μl of anti-CD44 v9 region PE-conjugated (red) for 10 min. An anti-PSA antibody was used as a negative control. (PDF) [file pone.0191109.s001.pdf]

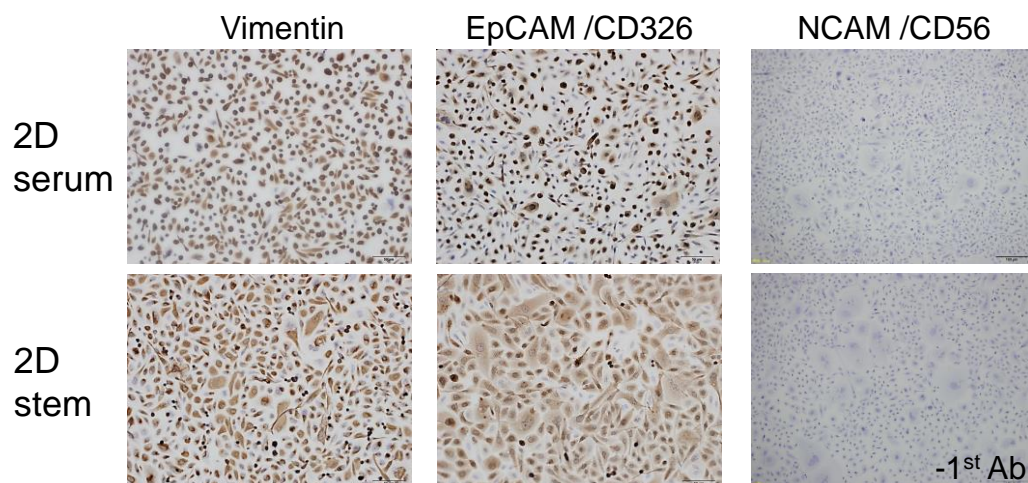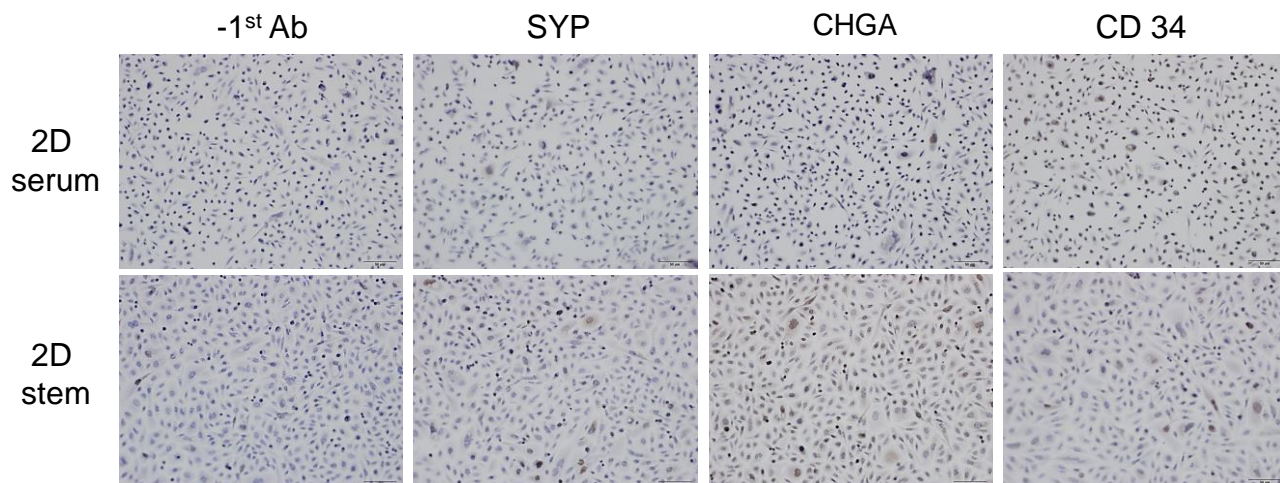

Supplement: S2 Fig — Percentages of positive cells were shown in Table 4. Photomicrographs were taken at a 20xmagnification. (PDF) [file pone.0191109.s002.pdf]
