# Supplementary material for: Organoids with cancer stem cell-like properties secrete exosomes and HSP90 in a 3D nanoenvironment
Source: PLoS One. 2018 Feb 7;13(2):e0191109. doi: 10.1371/journal.pone.0191109 (PMC5802492; doi:10.1371/journal.pone.0191109)
Supplement: S2 Table — Sequences of primers to detect and quantify cDNA levels of CD44 standard, CD44 variants, ESRP1/2, E-cadherin, CD133/PROM1, GAPDH, and β-actin were listed. (DOCX) [file pone.0191109.s004.docx]

**S1. List of primers.**

| Name | Sequence | Usage or product sizes |
| --- | --- | --- |
| h CD44 Fw standard exon 5 | 5’-GCACTTCAGGAGGTTACATC | CD44s/v amplification |
| h CD44 Rv standard exon 8 | 5’-ACTGCAATGCAAACTGCAAG | CD44s/v amplification |
| ACTB Fw | 5’-GGCATCCTCACCCTGAAGTA | Convensional PCR |
| ACTB Rv | 5’-GGGGTGTTGAAGGTCTCAAA | 30 cycles |
| h GAPDH Fw | 5’-GCCAAAAGGGTCATCATCTC | Realtime PCR |
| h GAPDH Rv | 5’-GTCTTCTGGGTGGCAGTGAT | 215 bp |
| h ESRP1 Fw | 5’-CAATATTGCCAAGGGAGGTG | Realtime PCR |
| h ESRP1 Rv | 5’-GTCCCCATGTGATGTTTGTG | 132 bp |
| h ESRP2 Fw | 5’-TGCCACAGAGGATGACTTTG | Realtime PCR |
| h ESRP2 Rv | 5’-ATTGACTGCTGGGCTCTTTG | 86 bp |
| Ecad/CDH1-h1937F/4815 | 5'-AGG AAT CCA AAG CCT CAG GT, | Realtime PCR |
| Ecad/CDH1-h2065R/4815 | 5'-TTG GGT TGG GTC GTTGTA CT | 128 bp |
| CD133/PROM1-h2855F/4257 | , 5'-TCGACCCCTTGAATTTGTTT, | Realtime PCR |
| CD133/PROM1-h2975R/4257 | 5'-TACACGTCCTCCGAATCCAT, | 120 bp |
| CD44s forward | 5'-GGAGCAGCACTTCAGGAGGTTAC, | Realtime PCR |
| CD44s reverse | 5'-GGAATGTGTCTTGGTCTCTGGTAGC, | 129 bp |
| CD44v9 forward | 5'-AGCAGAGTAATTCTCAGAGC, | Realtime PCR |
| CD44v9 reverse | 5'-TGATGTCAGAGTAGAAGTTGTT | 86 bp |
